# CUL3^LRB^ E3 ubiquitin ligases control thermosensory growth in *Arabidopsis* by differentially regulating HY5 and PIF4 protein stability

**DOI:** 10.1126/sciadv.aec7817

**Published:** 2026-03-06

**Authors:** Chirag Singhal, Gouranga Upadhyaya, Mohan Singh Rajkumar, Annayasa Modak, Vishmita Sethi, Shivani Singh, Devyan Das, Mukesh Jain, Sreeramaiah N. Gangappa

**Affiliations:** ^1^ Department of Biological Sciences, Indian Institute of Science Education and Research Kolkata, Mohanpur, 741246 West Bengal, India.; ^2^ School of Computational and Integrative Sciences, Jawaharlal Nehru University, New Delhi 110067, India.; ^3^ Centre for Climate and Environmental Studies, Indian Institute of Science Education and Research Kolkata, Mohanpur, 741246 West Bengal, India.

## Abstract

Rising temperatures from global warming harm plant health, reducing crop yields and threatening food security. Understanding the impact of climate change on plant health is crucial for developing climate-resilient crops. Here, we report LIGHT-RESPONSE BRIC-A-BRACK/TRAMTRACK/BROAD (LRB) E3 ubiquitin ligases as essential components of thermosensory growth in *Arabidopsis*. Single *lrb* mutants show subtle to moderate warm temperature insensitivity, while *lrb12* double and *lrb123* triple mutants exhibit complete insensitivity. Whole-genome transcriptomic analysis revealed that LRBs are crucial for the warm temperature–induced expression of genes involved in growth and hormone signaling. Genetic and gene expression analyses confirm that the LRBs are essential for PHYTOCHROME INTERACTING FACTOR 4 (PIF4)–mediated thermomorphogenesis. LRBs physically associate with PIF4, likely preventing its degradation and maintaining optimal protein levels. Concurrently, LRBs ubiquitinate and degrade the key repressor ELONGATED HYPOCOTYL5 during the daytime, thereby enhancing PIF4 signaling. Together, this study uncovers a dual mechanism by which LRBs potentiate thermosensory growth in response to warm ambient temperatures.

## Teaser

LRBs potentiate thermosensory growth by inhibiting HY5 activity while promoting PIF4 function.

## INTRODUCTION

Plants’ growth in a natural environment primarily depends on their ability to sense and integrate diverse environmental signals, such as light and temperature. Because plants lack locomotion, nature has equipped them with adaptive physiological tools that help them withstand ambient environmental changes. Plants undergo architectural modifications that promote efficient leaf cooling and may aid sustainable photosynthetic efficiency during exposure to warm temperatures (*1*–*4*). Plants grown at slightly elevated temperatures show accelerated vegetative to reproductive transitions and senesce comparatively early (*5*–*7*). A suite of morphological alterations that plants undergo in response to high ambient temperatures below the heat stress range is termed thermomorphogenesis (*7*, *8*). These alterations contribute to adaptive growth acclimation, helping the plant thrive in high ambient temperature conditions that would otherwise be detrimental (*9*). While warm temperatures promote plant growth and reproduction, they substantially affect immunity and crop yield, posing a threat to sustainable food security (*10*–*12*).

Uncovering the molecular mechanisms that mediate thermomorphogenesis is crucial to understanding how plants respond to subtle changes in climate, which is essential for developing climate-resilient crops. The basic helix–loop–helix (bHLH) transcription factor, PHYTOCHROME INTERACTING FACTOR 4 (PIF4), orchestrates transcriptome reprogramming in response to warm ambient temperatures to control various developmental and physiological responses (*4*, *13*–*15*). Elevated temperatures promote PIF4 gene expression and protein stabilization, enhancing its binding to target gene promoters (*13*, *16*–*18*). Under optimal temperature conditions, phytochrome B (phyB), a red-light–absorbing photoreceptor, negatively regulates PIF4 by degrading it in a red-light–dependent manner (*19*, *20*). The phyB also controls temperature sensing through HEMERA (HMR) and REGULATOR OF CHLOROPLAST BIOGENESIS (RCB). HMR promotes PIF4 accumulation, while RCB collaborates with HMR to stabilize PIF4 during the day, thereby facilitating warm temperature–induced gene expression (*21*, *22*). Moreover, HEAT SHOCK TRANSCRIPTION FACTOR A1d accumulates in response to warm temperatures and stabilizes PIF4 by interfering with its interaction with PHYB, thereby promoting thermomorphogenesis (*23*). The B-BOX (BBX) proteins, BBX24 and BBX25, also promote PIF4 protein accumulation both under dark and light phases in response to warm temperatures (*24*). CONSTITUTIVE PHOTOMORPHOGENIC1 (COP1), DE-ETIOLATED1 (DET1), or SUPPRESSOR OF PHYA-105 (SPA) also stabilizes the PIF4 protein, promoting thermomorphogenesis (*18*, *25*–*27*).

ELONGATED HYPOCOTYL5 (HY5), a basic-leucine zipper (bZIP) transcription factor, negatively regulates thermomorphogenesis by suppressing *PIF4* gene transcription and competing for binding to key cis-regulatory elements of temperature-regulated genes (*18*, *28*–*30*). HY5 promotes photomorphogenic development, including photosynthesis and cotyledon expansion, in contrast to PIF4’s promotion of skotomorphogenic growth (*18*, *29*, *31*–*36*). COP1, acullin-4 (CUL4)-based E3 ubiquitin ligase, targets HY5 directly for degradation in the dark both at optimal and warm temperatures (*27*, *37*). DET1, another CUL4-associated E3 ubiquitin ligase, promotes COP1-HY5 interactions and thus indirectly suppresses photomorphogenesis (*38*). SPA1 to SPA4 proteins also promote HY5 degradation via COP1 in the dark (*39*, *40*). Moreover, COP1 and SPA1 stabilize HY5 in response to ultraviolet-B light (*41*). This intricate regulatory network for HY5 suggests that the precise regulation of HY5 protein concentration is critical for promoting growth and development in response to varying light and temperature cues.

LIGHT-RESPONSE BRIC-A-BRACK/TRAMTRACK/BROAD (LRB) are E3 ubiquitin ligases that belong to a family of BRIC-A-BRAC, TRAMTRACK, and BROAD COMPLEX/POX VIRUS and ZINC FINGER proteins (*42*–*44*). LRBs control growth at various stages in the life cycle of *Arabidopsis*, including seed germination, hypocotyl growth, petiole elongation, cotyledon opening, rosette shape, and flowering time (*42*). They form Cullin 3–based ubiquitination complexes to degrade targets such as phyB and phyD via the ubiquitin-proteasome pathway (*42*). LRBs also degrade FRIGIDA to promote vernalization after cold stress (*45*). LRBs interact with cryptochrome 1(CRY1) and CRY2 in response to blue light and ubiquitinate them to control seedling photomorphogenesis and flowering (*46*, *47*). A recent study shows that TRANSPARENT TESTA GLABRA1 interacts with LRB2, further promoting the degradation of CRY1 (*48*).

It has been demonstrated that phyB recruits LRB E3 ubiquitin ligases to mediate the red-light–dependent degradation of PIF3 (*44*). Genetic and phenotypic analyses of double (*lrb1lrb2*) and triple (*lrb1lrb2lrb3*) mutants show a hyperphotomorphogenic response, characterized by short hypocotyls in red light (*42*, *44*), suggesting that LRBs act as positive regulators of hypocotyl elongation. The short hypocotyl phenotype of *lrb* mutants is proposed to result from elevated phyB protein levels (*44*). Warm ambient temperatures promote the conversion of phyB from its active form (Pfr) to its inactive form (Pr) (*49*), thereby activating PIF4 (*50*). Accordingly, one might expect that warm temperatures would alleviate the short hypocotyl phenotype in *lrb123* mutants. The hypocotyl lengths of *lrb12* and *lrb123* mutants remained unchanged between 22° and 27°C, indicating that these mutants do not respond to elevated temperatures. This led us to hypothesize that LRBs are key activators of growth crucial for thermosensory responses. Through detailed transcriptomic analysis, genetic interaction studies, and biochemical studies, we have demonstrated that LRBs are essential for promoting temperature-mediated growth in a PIF4-dependent manner. We further found that LRBs target HY5 for degradation through the ubiquitin-proteasome system (UPS).

## RESULTS

### LRBs are essential for warm ambient temperature–induced growth

**Fig. 1.**
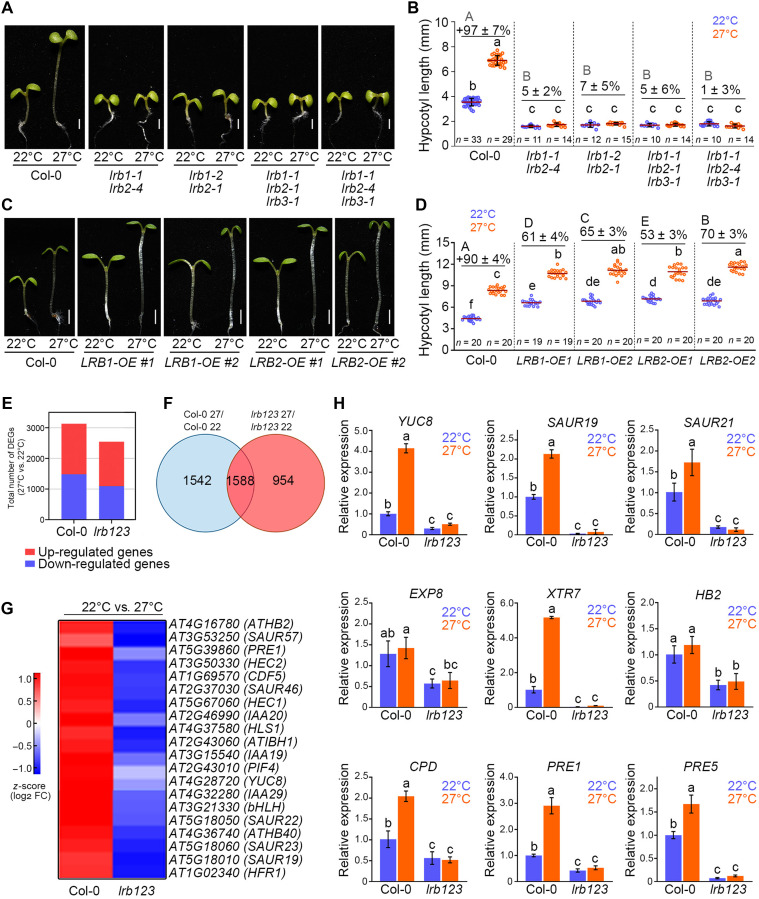
The *lrb123* mutants show strong defects in thermosensory growth. (**A** to **B**) Representative seedling images (A), hypocotyl length, and the percentage of hypocotyl growth (27°/22°C) response (B) of 6-day-old Col-0, *lrb12*, and *lrb123* mutants grown at 22°C and warm temperatures (27°C) under short-day (SD) photoperiod. Scale bars, 2 mm. (**C**) Representative seedling images of Col-0, *LRB1-OE*, and *LRB2-OE* lines grown under SD. Seedlings were grown as in (A). Scale bars, 2 mm. (**D**) Normalized hypocotyl length (27°/22°C) of the seedlings shown in (C). In (B) and (D), different small letters denote that the groups are significantly different from one another [one-way analysis of variance (ANOVA) followed by Tukey’s post hoc test; *P* < 0.05]. *n* indicates the number of seedlings. (**E** to **G**) Differentially regulated genes in Col-0 and *lrb123* in response to temperature (27°/22°C) in the 6-day-old seedlings (E). Venn diagram showing differentially regulated temperature-responsive genes shared between the *lrb123* mutant and Col-0 (F). Differentially regulated genes that are temperature inducible are compared via a heatmap in (G). A cutoff of log_2_ fold change ≥ 1 or < −1 with *P* < 0.05 was used to identify differentially expressed genes (DEGs). Three biological triplicates were analyzed. FC, fold change. (**H**) Relative expression of temperature-inducible genes measured through reverse transcription quantitative polymerase chain reaction (RT-qPCR) from 6-day-old seedlings of the indicated genotypes. *EF1*α was used as an endogenous control. Expression of genes in Col-0 at 22°C is used for normalization. The bar represents a mean ± SD of three biological replicates.

We first examined *lrb* mutants at a warm temperature of 27°C to characterize their thermosensory response. Comparing the hypocotyl growth at 22°C, we observed that *lrb1* and *lrb2* mutants exhibit shorter hypocotyls than the wild type (WT) (fig. S1A), and at 27°C, they exhibit reduced hypocotyl elongation in response to the warm temperature (fig. S1B). Analyzing the *lrb1* and *lrb2* mutant phenotypes, it was also evident that LRB2 plays a greater role in regulating hypocotyl elongation. In the *lrb3* mutant, there is only a subtle difference in hypocotyl length at 22°C or suppression of temperature response at 27°C as compared to that in the WT (Col-0), suggesting that LRB3 does not contribute or have a minor role in regulating hypocotyl growth and thermosensory responses. To test the possibility of their function being additive in hypocotyl elongation and thermosensory response, we repeated the experiments with double *lrb1 lrb2* (now onward denoted as *lrb12*) and triple *lrb1 lrb2 lrb3* (now onward denoted as *lrb123*) mutants and compared them with the Col-0 (Fig. 1, A and B). Notably, in the *lrb12* mutant lines, a substantial reduction in hypocotyl length was observed, with hypocotyls measuring less than those of the Col-0, *lrb1*, or *lrb2* mutant. The shorter hypocotyl length of *lrb12* than *lrb1* or *lrb2* alone suggests that *lrb1* and *lrb2* are additive in promoting hypocotyl growth in white light. At 27°C, our data indicate that *lrb12* becomes insensitive to warm temperatures, completely suppressing temperature-induced hypocotyl elongation, as hypocotyl lengths are comparable at 22° and 27°C. However, by crossing *lrb3* with the *lrb12* mutant, we observed no substantial differences in hypocotyl length or temperature response in the *lrb123* as compared to the *lrb12* mutant, strengthening our previous observation that *lrb3* has only a minor effect on hypocotyl growth. On further exploration, the hypocotyl anatomy revealed that *lrb123* mutants have smaller cells than the WT at both 22° and 27°C throughout the middle length of the hypocotyl (fig. S1, C and D), suggesting that the short hypocotyl phenotype of *lrbs* is due to reduced cell elongation.

We also generated overexpression lines of *LRB1* and *LRB2* and found that they displayed elongated hypocotyls compared to Col-0 at 22° and 27°C (Fig. 1, C and D). However, their percentage hypocotyl growth (27°/22°C) was reduced compared to Col-0. To investigate the functional relevance of LRBs under warm temperatures, we checked their protein levels. Our immunoblotting results reveal that LRB1 and LRB2 are more abundant at warm temperatures as detected using anti-Myc antibody (fig. S2A). Moreover, the levels of both LRB1 and LRB2 proteins were observed to increase gradually when seedlings initially grown at 22°C were shifted to 27°C (fig. S2, B and C), highlighting the temperature-responsive nature of LRBs.

Furthermore, for the functional characterization of the *lrb123* mutant, we first delve into the transcriptome of Col-0 and *lrb123* and conduct differential expression studies by comparing gene expression profiles at 22° and 27°C (dataset S1). We observed that a total of 3130 genes was differentially regulated in Col-0, whereas *lrb123* shows a total of only 2542 genes differentially regulated in response to temperature (Fig. 1E). Among the differentially expressed genes (DEGs), the *lrb123* mutant shares 1588 temperature-responsive genes with Col-0 (Fig. 1F). Of these genes, we identified multiple temperature-inducible genes that exhibit down-regulation in the *lrb123* mutant in response to temperature (27°/22°C), compared to Col-0, as evident from the *z*-score in (Fig. 1G).

Consistent with the transcriptomic data, validation through the reverse transcription quantitative polymerase chain reaction (RT-qPCR) analysis confirmed the down-regulation of key genes involved in temperature response. Many of these genes are involved in auxin biosynthesis. For instance, the expression of auxin biosynthetic gene *YUCCA8* (*YUC8*) was down-regulated in the *lrb123* compared to Col-0 at both 22° and 27°C (Fig. 1H). The *SMALL AUXIN UP RNA* (*SAUR*) genes play a crucial role in the auxin-mediated regulation of plant growth and development, responding to light and temperature signals. Specifically, *SAUR19 to SAUR24* genes are activated by warmer temperatures (*17**,* *51**–**53*). Notably, RT-qPCR results revealed significant induction of these genes at 27°C compared to 22°C in Col-0 seedlings. In comparison, their expression was severely reduced in the *lrb123* mutant at 22° and 27°C (Fig. 1G). Auxin primarily promotes elongation by enhancing the expression of cell-wall loosening genes such as *EXPANSIN 8 (EXP8)* and *XYLOGLUCAN ENDOTRANSGLYCOSYLASE 7 (XTR7)*. The RT-qPCR analyses revealed significant impairment in the expression of *EXP8* and *XTR7* in the *lrb123* mutant at 22° and 27°C compared to Col-0; however, these genes exhibit robust up-regulation in Col-0 in response to 27°C (Fig. 1G). Similarly, *ATHB2*, another auxin-responsive gene involved in cell elongation, is down-regulated in the *lrb123* (Fig. 1G). Furthermore, genes involved in brassinosteroid (BR) biosynthesis, such as *CPD* and signaling genes *PRE1* and *PRE5*, exhibited elevated expression in Col-0 at 27°C compared to 22°C and at the same time showed robust down-regulation in the *lrb123* mutant compared to Col-0, both at 22° and 27°C (Fig. 1G).

### LRBs are necessary for auxin homeostasis

**Fig. 2.**
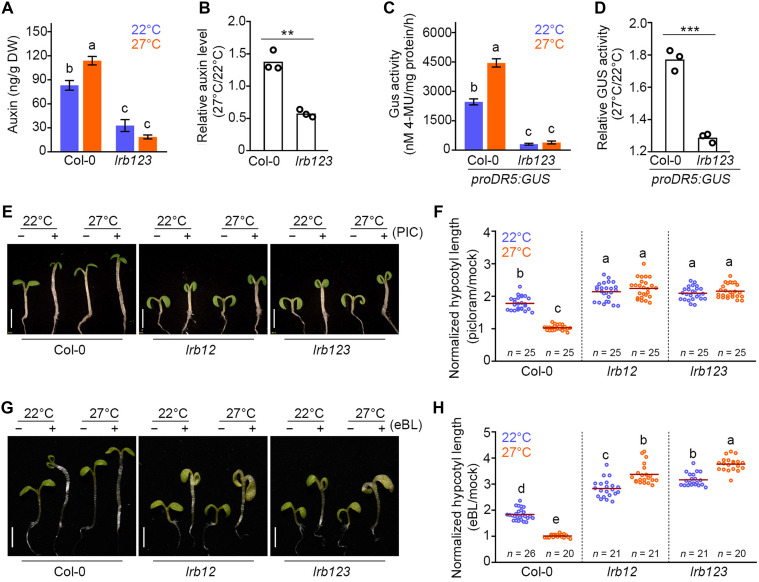
The *lrb* mutants are deficient in auxin accumulation and auxin signaling. (**A**) Auxin concentration in nanograms per gram of dry weight (DW) in 6-day-old seedlings (*n > *3**). (**B**) Relative IAA content in the seedlings at 27°/22°C (*n =* 3 biological replicates). (**C**) Auxin reporter *proDR5:GUS* promoter-reporter activity as revealed by fluorometric quantification assay in 6-day-old seedlings of Col-0 and *lrb123* mutant grown at optimum (22°C) and warm temperature treatment (27°C). h, hour. 4-MU, 4-methylumbelliferone. (**D**) Relative *proDR5:GUS* activity (27°/22°C) in Col-0 and *lrb123* mutant seedlings (*n >* 3 biological replicates). (**E**) Six-day-old seedlings of Col-0, *lrb12*, and *lrb123* grown in the presence or absence [dimethyl sulfoxide (DMSO) as mock] of 5 μM picloram at 22° and 27°C (scale bars, 2 mm). PIC, picloram. (**F**) Normalized hypocotyl length (picloram/mock) in Col-0, *lrb12*, and *lrb123* mutants. Seedlings from two independent experiments were measured. (**G**) Six-day-old seedlings of Col-0, *lrb12*, and *lrb123* seedlings grown in the presence or absence of 5 μM eBL (ethanol as mock) at either 22° or 27°C (scale bars, 2 mm). (**H**) Normalized hypocotyl length (eBL/mock) in Col-0, *lrb12*, and *lrb123* mutants. Seedlings from two independent experiments were measured. (A), (C), (F), and (H) denote that the groups are significantly different from one another (one-way ANOVA followed by Tukey’s post hoc test; *P* < 0.05). *n* indicates the number of seedlings measured. In (B) and (D), the *P* value was calculated by an unpaired, two-tailed Student’s *t* test. In (F) and (H), *n* indicates the number of seedlings measured. Seeds were stratified for 4 days and then allowed to germinate for 2 days at 22°C before being transferred to 22° or 27°C.

Auxins play a pivotal role in hypocotyl elongation (*54**–**56*). Elevated temperature promotes auxin accumulation (*57*). We hypothesized that the short hypocotyl phenotype of *lrb* mutants could be due to reduced auxin biosynthesis or perturbed auxin signaling. To test this, we quantified the free auxin levels in the *lrb123* mutant and WT (Col-0) seedlings. We found a significant increase in free auxin [indole-3-acetic acid (IAA)] levels in Col-0 seedlings in response to warmer temperatures (Fig. 2A). In contrast, auxin levels were significantly reduced in *lrb123* mutants compared to WT seedlings at both 22° and 27°C, suggesting that *lrb123* mutants have reduced endogenous auxin levels (Fig. 2A). Moreover, the reduced relative auxin levels (27°/22°C) suggest that *lrb123* is less responsive to temperature-promoted elevation of auxin levels (Fig. 2B). To further assess this, we used a known auxin marker, a synthetic *DR5* promoter fused to a *Glucuronidase* (GUS) reporter (*pDR5:GUS*) transgenic line. GUS staining was first carried out to analyze the auxin distribution in the *lrbs123* seedling by comparing it with the WT. We observed proper GUS staining in the cotyledons of the Col-0 seedlings at 22°C (fig. S3A), whereas negligible staining was observed in the *lrb123* mutant. At 27°C, intense staining in the hypocotyl of the Col-0 seedlings was observed, suggesting that warm temperature directs enhanced auxin activity toward the hypocotyl. However, no staining was observed in the hypocotyls of *lrb123*. Quantitative measurement of GUS activity revealed that the *DR5* promoter activity was markedly reduced both at 22° and 27°C in the *lrb123* mutant background compared to Col-0 (Fig. 2C), confirming that the *lrb123* mutant is indeed deficient in endogenous auxin levels. Moreover, the reduced relative GUS activity in *lrb123* (27°/22°C) compared to the Col-0 confirms that LRBs are required for temperature-induced elevation of auxin levels (Fig. 2D).

**Fig. 3.**
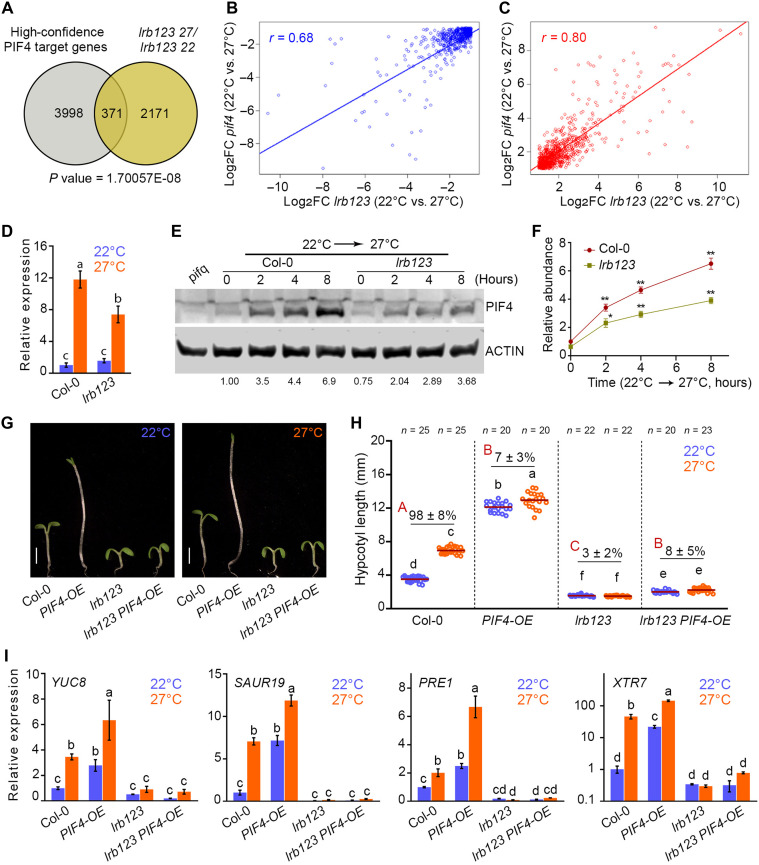
PIF4-mediated thermomorphogenesis requires LRBs. (**A**) DEGs in *lrb123* (27°/22°C) overlap significantly with the genes that are targeted by PIF4 as reported . (**B** and **C**) Relation between down-regulated (B) and up-regulated (C) DEGs in 6-day-old seedlings of *lrb123* and *pif4* mutants in response to warm temperature. The scatter plots and regression line show the correlation in *r*. In (A), the *P* value was calculated using the hypergeometric test. In (A) to (C), a cutoff of log_2_ fold change ≥ 1 or < −1 with *P* < 0.05 was used to identify DEGs. Three biological triplicates were analyzed. (**D**) RT-qPCR analysis of the relative *PIF4* gene expression in 6-day-old seedlings. *EF1*α was used as an endogenous control. The bar represents the mean ± SD (*n =* 3 biological replicates). (**E** and **F**) Immunoblots showing PIF4 protein (E) and the relative protein levels (F) in Col-0 and *lrb123* mutants grown at 22°C and then shifted to 27°C. The data shown represent the mean ± SD of three biological replicates. ACTIN is used as a loading control. The relative intensity of PIF4/ACTIN is calculated and normalized by the same at the 0-hour time point in WT after transferring to 27°C, as mentioned below the immunoblots. Asterisks denote statistical significance (Student’s *t* test; *P < 0.05 and **P < 0.01) in the PIF4 protein level from the 0 time point in the Col-0. (**G** to **I**) Representative seedling pictures (G), hypocotyl length and percentage of hypocotyl growth (H), and RT-qPCR analysis of thermoresponsive genes expression (I) of 6-day-old seedlings of the indicated genotypes grown in 22° and 27°C under SD. Scale bars, 2 mm. The small and capital letters denote the statistically significant differences (one-way ANOVA followed by Tukey’s post hoc test; *P* < 0.05). *EF1*α was used as an endogenous control. Expression of genes in Col-0 at 22°C is used for normalization. The bar represents the mean ± SD (*n =* 3 biological replicates).

As our above data suggested, the short hypocotyl phenotype of *lrb123* is due to defective auxin levels; the exogenous application of auxins should rescue it. To test this, we grew *lrb* mutants on Murashige and Skoog (MS) medium supplemented with picloram (a synthetic auxin) and compared the hypocotyl growth of these seedlings with that of mock-treated seedlings at 22° and 27°C. The *lrb* single mutants, *lrb1*, *lrb2*, and *lrb3*, displayed a nearly twofold increase in hypocotyl length in response to picloram at 22°C compared to the mock, which is comparable to that of Col-0 (fig. S3, B and C). At 27°C, we did not see much difference in the Col-0 and *lrb3* hypocotyl length in response to picloram, whereas there was a significant increase in hypocotyl lengths of *lrb1* and *lrb2* mutants (fig. S3C). However, the extent of response is reduced at 27°C compared to 22°C (fig. S3D). In *lrb12* and *lrb123* mutants, we also observed a twofold increase in hypocotyl lengths at 22°C in response to picloram compared to mock (Fig. 2, E and F). In addition, a twofold increase in hypocotyl length was also observed at 27°C in *lrb12* and *lrb123* mutants (Fig. 2, E and F), again supporting our hypothesis that LRBs are additive in thermosensory response. BRs play a crucial role in promoting temperature-induced hypocotyl elongation and act downstream of auxin signaling to facilitate thermomorphogenesis (*28**,* *58*). When we grew seedlings on growth medium supplemented with epibrassinolide (eBL), the Col-0 seedlings responded significantly to eBL compared to the mock, displaying a nearly twofold change at 22°C, whereas seedlings grown at 27°C did not show a significant response to eBL (Fig. 2, G and H, and fig. S4). Adding eBL to growth medium effectively rescued the moderate hypocotyl phenotype of *lrb* single mutants, displaying a nearly twofold change such as Col-0 (fig. S4). The short hypocotyl phenotype of *lrb12* double and *lrb123* mutants was significantly rescued, as they showed a robust response greater than threefold to eBL at both 22° and 27°C, compared to Col-0 (Fig. 3, G and H), underscoring the role of BR in mitigating the *lrb* mutant phenotypes. Although exogenous application of auxin and BR greatly rescued the short hypocotyl phenotype of *lrb* mutants at 22°C, the absolute hypocotyl lengths of *lrb12* and *lrb123* mutants were still shorter than those of Col-0 at 27°C (Fig. 2, E and G). This suggests that the short hypocotyl phenotypes of these mutants are partly due to the defective auxin/BR metabolism and signaling.

### PIF4-mediated thermosensory growth requires LRBs

PIF4 is a key regulator of the thermomorphogenesis (*4*, *13*–*15*). The thermo-insensitive phenotype of *lrb12* and *lrb123* mutants phenocopies the *pif4* mutant (*59*). The RNA sequencing (RNA-seq) and RT-qPCR analysis of the *lrb123* transcriptome in response to temperature suggest that temperature-responsive genes are misregulated in the absence of LRBs compared to the Col-0 control. PIF4, as a transcription factor, regulates the expression of growth-related genes in response to temperature (*13**,* *16**–**18*). Using our transcriptome data, we first examine the overlap between the misregulated genes in the *lrb123* mutant in response to temperature (22°/27°C) and the genes targeted by PIF4 (Fig. 3A) (*60*). We found a significant overlap (*P* = 1.70057 × 10^–8^) with 371 common genes in the sets of genes targeted by PIF4 and the genes misregulated in the *lrb123* mutant in response to temperature (Fig. 3A). We then analyzed the *pif4* mutant transcriptome in response to temperature to understand if they share a similar pattern of misregulated genes. A total of 2335 genes was found to be differentially regulated in the *pif4* mutant transcriptome in response to temperature. Notably, we observed a significant overlap of 1417 genes misregulated in the *lrb123* and *pif4* mutant transcriptomes in response to temperature (fig. S5, A and B). Upon comparing the subsets of coregulated genes between *lrb123* and *pif4*, we found a relationship coefficient of *r* = 0.68 among the commonly down-regulated genes (Fig. 3B) and *r* = 0.8 among the commonly up-regulated genes (Fig. 3C) in response to warm temperature compared to optimal temperature (27°/22°C).

Among the widely down-regulated genes in *lrb123* and *pif4* mutants, we found a subset of genes targeted by PIF4 that was comparatively up-regulated in Col-0 (fig. S5C). Notably, these genes are integral to the biosynthesis and signaling pathways of auxin and BRs, which promote cellular elongation and growth. On the contrary, another set of genes targeted by PIF4 was identified among the commonly up-regulated genes in *lrb123* and *pif4* mutants that were down-regulated in Col-0 in response to temperature (fig. S5D). Markedly, these genes are involved in light signaling and promote photomorphogenesis. To examine the functional relevance of the DEGs, we performed Gene Ontology (GO) enrichment analysis for the sets of up-regulated or down-regulated genes under warm conditions (27°C) in the Col-0, *lrb123*, and *pif4* mutants (dataset S2 and fig. S6). Our findings revealed robust enrichments of GO terms “response to stimulus” in up-regulated and down-regulated gene sets, consistently observed across the Col-0, *lrb123*, and *pif4* mutants (fig. S6). The GO category “response to stress” was also enriched among the differentially regulated genes in all sets, particularly among the down-regulated genes in *lrb123*, indicating a specialized regulatory role of LRBs in stress-related pathways. Moreover, the GO term “heat acclimation” was exclusively enriched among up-regulated genes in the *lrb123* mutant, emphasizing the pivotal role of LRBs in orchestrating genes involved in heat acclimation processes (fig. S6). In addition, we identified specific GO enrichments that were consistently observed in both *lrb123* and *pif4* mutants but not in Col-0. For instance, the up-regulated gene sets were significantly enriched for terms related to “secondary metabolic processes” and “regulation of transcription,” suggesting that LRBs and PIF4 may play suppressive roles in secondary metabolite pathways under warm conditions (fig. S6). Conversely, GO categories such as “cellular cell-wall organization” were enriched among down-regulated genes (fig. S6), indicating a potential role of these factors in promoting cell-wall organization under elevated temperatures.

To determine whether LRBs can influence *PIF4* gene expression or protein abundance, we first conducted gene expression analysis for *PIF4* in the *lrb123* mutant and compared it with the WT. In line with our RNA-seq results (Fig. 1G), qPCR results confirmed that *PIF4* is down-regulated in the *lrb123* mutant (Fig. 3D). To investigate protein levels in the *lrb123* mutant, we performed immunoblot analysis. Our results suggest that PIF4 is more stable in the Col-0 at 27°C compared to 22°C (fig. S7); however, the levels of PIF4 protein in the *lrb123* mutant were low compared to the Col-0 at both 22° and 27°C, suggesting that LRBs are required for optimum gene expression and protein abundance of PIF4. To further substantiate the role of LRBs in promoting PIF4 function at warm temperatures, we grew the Col-0 and *lrb123* mutant seedlings at 22°C for 6 days and shifted them to 27°C. A gradual increase in PIF4 protein levels was observed in Col-0, reaching a sevenfold increase in ~8 hours after shifting to 27°C (Fig. 3, E and F). However, in the *lrb123* mutant, the increment is significantly reduced compared to the Col-0 (Fig. 3, E and F), underscoring the importance of LRBs for optimal PIF4 protein abundance.

To study the genetic interaction between *LRBs* and *PIF4*, we introduced a *pPIF4:PIF4-FLAG* (*18*) transgene that overexpresses PIF4 (hereafter *PIF4-OE*) into the *lrb123* mutant, as well as *LRB* overexpression backgrounds. Measuring hypocotyl lengths of *PIF4-OE* and *lrb123 PIF4-OE* seedlings under 22° and 27°C conditions revealed that the *PIF4-OE* line exhibits a hypersensitive hypocotyl elongation phenotype, showing longer hypocotyls even at 22°C compared to the WT, as expected. However, under the *lrb123* mutant background, the absence of LRBs strongly mitigates the exaggerated hypocotyl phenotype of *PIF4-OE*, evident from the *lrb123 PIF4-OE* phenotype, at 22°C (Fig. 3, G and H). In addition, the hypocotyl lengths of *lrb123 PIF4-OE* at 22° and 27°C were comparable, suggesting that the absence of LRBs is epistatic to *PIF4-OE*. At the same time, *PIF4-OE LRB1-OE* or *PIF4-OE LRB2-OE* double transgenic lines displayed further elongated hypocotyls compared to the *PIF4-OE* alone at both 22° and 27°C (fig. S8, A and B).

Consistent with the strong suppression of *PIF4-OE* hypocotyl phenotype by *lrb123*, RT-qPCR analysis indicated that PIF4 target genes such as *YUC8*, *SAUR19*, *PRE1*, and *XTR7* were down-regulated in the *PIF4-OE lrb123* background compared to the *PIF4-OE* or the Col-0 at both 22° and 27°C (Fig. 3F). The expression of light-inducible genes, such as *RBCS1A*, *CAB1*, *ELIP2*, and *SAUR14*, was found to be typically low in the *PIF4-OE* line at both 22° and 27°C (fig. S9). However, the expression of these genes was elevated in *lrb123 PIF4-OE* at 22°C and more prominently at 27°C (fig. S9). Supporting the gene expression data, our immunoblotting data also suggest that PIF4 protein levels are considerably reduced in the *lrb123 PIF4-OE* background compared to the *PIF4-OE* line, both at 22° and 27°C under short-day (SD) conditions (fig. S10A), implying that LRB activity is essential for maintaining optimal PIF4 protein levels and thereby facilitating the robust activation of thermomorphogenic growth.

Studies of E3 ubiquitin ligases have revealed that, in addition to mediating substrate degradation via the ubiquitin-proteasome pathway, some E3 ligases also stabilize the transcription factors through direct physical interactions (*61**–**63*). Given that LRBs are essential for the optimal PIF4 protein accumulation, we hypothesized that LRBs likely stabilize PIF4 through a noncanonical mechanism involving physical association. To test this possibility, we conducted protein-protein interaction studies. In a yeast two-hybrid assay, PIF4 was fused to the galactose-responsive transcription factor (GAL4) DNA binding domain (DBD) and used as bait, while full-length LRB1 and LRB2 were fused to the GAL4 activation domain (AD) as prey. Cotransformants expressing PIF4 with either LRB1 or LRB2 displayed normal growth on selective SD medium lacking Leu, Trp, His, and Ade (−LWHA) supplemented with 3-aminotriazole (3-AT), indicating positive interactions (fig. S10B). In contrast, no growth was observed in empty vector combinations (fig. S10B). The interactions between LRB1 and LRB2, as well as between T-antigen and p53, serve as positive controls (fig. S10B). These interactions were further examined in planta via bimolecular fluorescence complementation (BiFC) assay in onion epidermal cells. Coexpression of either N-terminal yellow fluorescent protein (nYFP)–LRB1 or nYFP-LRB2 with C-terminal yellow fluorescent protein (cYFP)–PIF4 resulted in strong nuclear YFP fluorescence (fig. S10C), whereas no fluorescence was observed in the corresponding negative controls using empty vector combinations (fig. S10C). To further substantiate these findings, we performed in vivo coimmunoprecipitation (Co-IP) assays using 6-day-old seedlings of *LRB1-Myc* and *LRB2-Myc* overexpression lines. When we immunoprecipitated LRB1-Myc or LRB2-Myc proteins using anti-Myc antibody, we could detect PIF4 in the immunoprecipitated fractions of both the *LRB1-OE* and *LRB2-OE* lines, while no signal for PIF4 was observed in Col-0 (WT) samples (fig. S10D). Together, these genetic and biochemical data demonstrate that LRBs physically associate with PIF4, likely preventing its degradation and promoting the accumulation of PIF4 protein.

### LRBs genetically interact with HY5 and inhibit its function

**Fig. 4.**
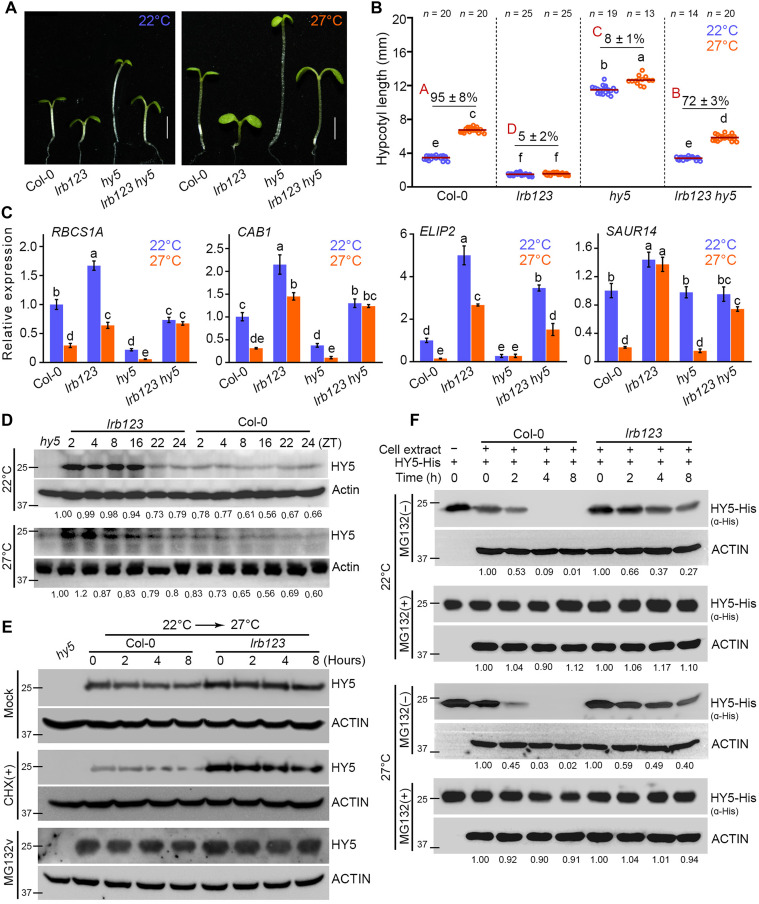
LRBs genetically interact and suppress HY5 function. (**A**) Representative seedling images (A) and hypocotyl length and percentage of hypocotyl growth (**B**) of 6-day-old seedlings of indicated genotypes grown at 22° and 27°C under SD. Scale bars, 2 mm. Small and capital letters respectively denote the significant differences (one-way ANOVA followed by Tukey’s post hoc test; *P* < 0.05) in hypocotyl length and percentage of hypocotyl growth. *n* indicates the number of seedlings measured. (**C**) RT-qPCR analysis of relative expression of temperature-repressible genes in 6-day-old seedlings of the indicated genotypes. *EF1*α was used for normalization. The bar shows the mean ± SD for *n =* 3 biological replicates. (**D**) HY5 protein abundance in the 6-day-old seedlings of Col-0 and *lrb123* mutant grown at 22° or 27°C. ACTIN is used as a loading control. The relative intensity of HY5/ACTIN is calculated and normalized by the same at Zeitgeber 2 (ZT2) in WT at 22°C, as shown below the immunoblots. (**E**) HY5 protein abundance in the 6-day-old seedlings of the indicated genotypes. Seedlings grown at 22°C were treated with mock, cycloheximide (CHX), or *N*-carbobenzyloxy-l-leucyl-l-leucyl-l-leucinal (MG132) and were either retained at 22°C or shifted to 27°C. Seedling samples were harvested at the indicated time points (after transferring to 27°C). The anti-HY5 antibody was used to detect the HY5 protein. ACTIN levels serve as a loading control. (**F**) Cell-free degradation of recombinant HY5 protein in the presence or absence of LRBs. HY5-His (40 ng) protein was incubated with protein extract from 22° and 27°C grown seedlings, at 22° and 27°C. Purified recombinant HY5-His (40 ng) from *Escherichia coli* was incubated with 160 ng of seedling extract at 22° or 27°C. Separate reactions were supplemented with mock or MG132 treatment. HY5-His was detected with anti-His antibody (Invitrogen). ACTIN is used as a loading control for plant extract. The relative intensity of HY5-His/ACTIN is calculated and normalized by the same at respective 0-hour time points in Col-0 and *lrb123* mutant, as shown below the immunoblots.

HY5 is a critical negative regulator of thermomorphogenesis, inhibiting the expression of growth-promoting genes by competing with PIF4 for binding at the promoter sites of target genes and suppressing their transcription (*18**,* *28**,* *29*). To confirm this, we first compared the genes regulated by PIF4 (*60*) and HY5 (*36*). We observed a significant overlap of 1402 genes that were common targets in both (fig. S11A). From this set of common target genes of PIF4 and HY5, we found a significant overlap (*P* = 0.003527) with DEGs in the *lrb123* mutant (fig. S11B). Our gene expression data from both RNA-seq and RT-qPCR revealed that key marker genes of photomorphogenesis are strongly derepressed in the *lrb123* mutant background at 22° and 27°C (fig. S9). Notably, many of these genes, such as *CAB1*, *RBCS1A*, and *ELIP2*, are direct targets of HY5 (*35**,* *64*). These findings indicated that HY5 activity is likely enhanced in the *lrb123* mutant background, implying a possible functional connection. To test this at the genetic level, we generated *lrb123 hy5* quadruple mutant to assess the genetic interactions between LRBs and HY5. We found that *hy5* can significantly suppress the short hypocotyl phenotype of the *lrb123* mutant (Fig. 4, A and B). The hypocotyl in the *lrb123 hy5* mutant was much longer compared to *lrb123* and is comparable to Col-0 at 22° and 27°C (Fig. 4, A and B). In line with the suppression of the short hypocotyl phenotype of *lrb123* by *hy5*, the RT-qPCR analysis revealed that temperature-induced growth-responsive genes such as *YUC8*, *SAUR19*, *PRE1*, and *XTR7* were up-regulated in the *lrb123 hy5* compared to the *lrb123* mutant (fig. S12).

Furthermore, we performed gene expression analysis of the key light signaling genes in the *lrb123 hy5* mutant and compared them with those of the *lrb123* mutant. The expression of these light-responsive genes, such as *RBCS1A*, *CAB1*, *ELIP2*, and *SAUR14*, was suppressed in the *lrb123 hy5* compared to *lrb123* (Fig. 4C). As anticipated, these genes exhibit marked down-regulation in the *hy5* mutant compared to Col-0 at 22° and 27°C, indicating that LRBs are antagonistic to HY5 function (Fig. 4C). HY5 has been shown to inhibit *PIF4* gene expression in response to warm temperatures (*28*). The RT-qPCR data revealed a significant reduction in the *PIF4* transcript levels in the *lrb123* mutant background compared to Col-0 at 27°C but not at 22°C (Fig. 3D and fig. S13). In contrast, *PIF4* expression was slightly elevated in the *hy5* mutant at 27°C (fig. S13); however, in the *lrb123 hy5* mutant, the *PIF4* expression was comparable to Col-0 (fig. S13), suggesting that the reduced *PIF4* expression in the *lrb123* mutant is at least partially due to elevated HY5 protein levels. The rescuing of the short hypocotyl of the *lrb123* mutant suggests that hypocotyl length, temperature insensitivity, and gene expression phenotypes in the *lrb123* mutant could be manifested because of the enhanced HY5 activity, implying that LRBs likely inhibit HY5 to promote PIF4-mediated thermomorphogenesis.

### LRBs degrade the HY5 transcription factor, likely through the 26*S* proteasomal pathway

To test whether LRBs have any role in regulating the HY5 protein stability, we analyzed the dynamics of HY5 protein abundance in Col-0 and *lrb123* mutant using native HY5 antibodies (Fig. 4D). We found that HY5 protein accumulation is markedly higher in the *lrb123* mutant than in Col-0, both at 22° and 27°C at all the time points (Fig. 4D). Notably, HY5 is more stable in *lrb123* during the day at 22°C [Zeitgeber 2 (ZT2) to ZT8] and at the beginning of the night (ZT16) (Fig. 4D). HY5 levels at 27°C were strongly elevated during the day (ZT2 to ZT8) compared to the WT, suggesting that LRBs exert stronger inhibition on HY5 at warm temperatures. This is likely due to elevated levels of both LRB1 and LRB2 proteins at 27°C compared to 22°C (fig. S2). LRBs are E3 ubiquitin ligases that target the substrates for ubiquitination and subsequent degradation through the UPS (*44*, *47*). Next, we wanted to investigate whether LRBs promote HY5 degradation via the UPS. To investigate this, we first checked the protein abundance of HY5 in the WT and *lrb123* mutant. Six-day-old seedlings of the WT and *lrb123* mutant, grown at 22°C, were treated with *N*-carbobenzyloxy-l-leucyl-l-leucyl-l-leucinal (MG132; a proteasome inhibitor) or cycloheximide (CHX; a translation inhibitor) and transferred to 27°C. We found that the HY5 protein was relatively stable in the mock, MG132-treated, or CHX-treated seedlings of *lrb123* mutant (Fig. 4E); however, in the Col-0, HY5 protein abundance is gradually reduced in the mock and more so in the CHX-treated samples (Fig. 4E), suggesting that reduced HY5 protein abundance is due to the degradation of the HY5 protein via proteasome system.

We performed an in vitro cell-free degradation assay to further substantiate these results. We expressed and purified the recombinant HY5 protein, tagged with 6× Histidine (HY5-6xHis), in *Escherichia coli* (BL21 strain) and performed a cell-free degradation assay. Recombinant HY5-His protein was added to total protein extracts from 6-day-old seedlings of Col-0 and *lrb12* mutant grown at 22° and 27°C, and the reactions were incubated under respective conditions. The protein stability at different incubation times was monitored by immunoblotting using anti-His antibodies. In the presence of total protein extract from Col-0 (WT), HY5-His levels decreased significantly after 2 hours of incubation, becoming nearly undetectable at 4- and 8-hour time points (Fig. 4F, first panel). However, when the protein extract was added with MG132, a 26*S* proteasomal inhibitor, HY5-His degradation was significantly inhibited throughout the incubation period (Fig. 4F, first panel), suggesting that HY5 protein degradation occurs via the 26*S* proteasome pathway as reported (*37*). Notably, the levels of HY5-His protein were higher in extracts prepared from the *lrb123* mutant seedlings than in Col-0. We observed that the HY5-His protein was stable even after 8 hours of incubation at 22° and 27°C, both in the *lrb123* protein extract (Fig. 4F). At the same time, in the presence of MG132, HY5-His stability was comparable to that in Col-0. These results confirmed that LRBs promote HY5 degradation through the 26*S* proteasomal pathway.

### LRBs physically interact with HY5 via its C-terminal domain

**Fig. 5.**
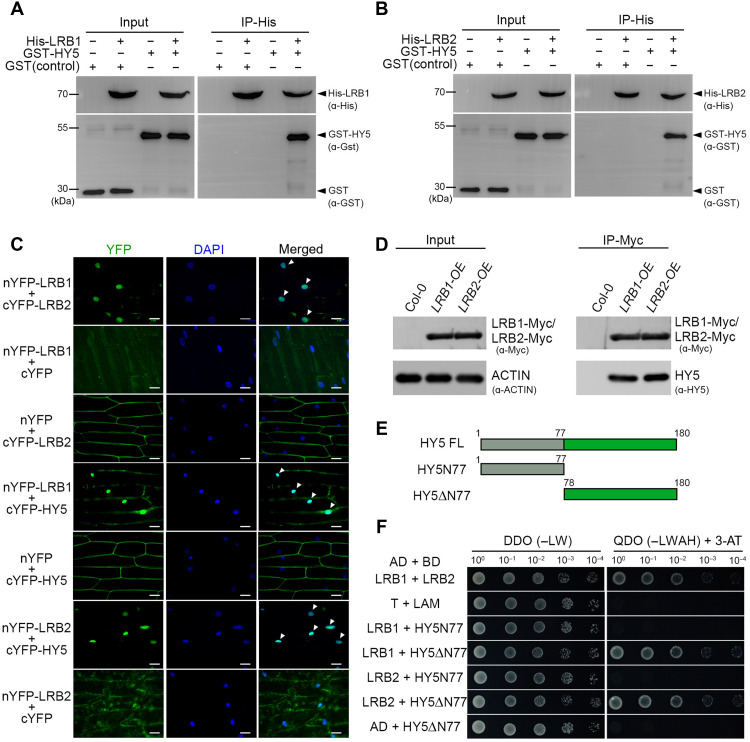
LRBs interact with HY5 and promote its ubiquitination. (**A** and **B**) In vitro pull-down assay showing LRB1 (A) and LRB2 (B) interact with HY5. IP was performed with Ni-NTA agarose beads. The IP (LRB1/LRB2) and Co-IP (HY5) products were detected in the coimmunoprecipitated samples using anti-His and anti-GST antibodies, respectively. The GST protein was used as a negative control. (**C**) Epifluorescence microscopy images showing BiFC signals of the indicated protein pairs transiently expressed in *Allium cepa* epidermal cells. Hoechst 33342 was used to stain the nuclei (green), and green fluorescent protein (red) signals are shown. Scale bars, 5 μm. The weak cytoplasmic YFP signal observed in some controls reflects background fluorescence. DAPI, 4′,6-diamidino-2-phenylindole. (**D**) Immunoblots showing Co-IP assay of HY5 protein with LRB1-Myc and LRB2-Myc. LRB1-Myc/LRB2-Myc were immunoprecipitated using anti-Myc antibody. The anti-HY5 antibody was used to detect coimmunoprecipitated HY5. Col-0 is used as a negative control. (**E**) Diagram illustrating the domain architecture of HY5 and its truncated versions. FL, full length. (**F**) Yeast two-hybrid assay showing interactions of LRB1 and LRB2 with truncated versions of HY5. Lam, Lamin; T, T-antigen. The left panel shows the growth of the cotransformed yeast Y2HGold strain on double-dropout (−Leu and −Trp) medium, and the right panel shows the growth on quadruple-dropout (−Leu, −Trp, -His, and −Ade) medium supplemented with 5 mM 3-AT.

Next, we aimed to investigate whether LRB-mediated degradation of the HY5 protein requires a direct physical interaction between LRB1/LRB2 and the HY5 protein. We performed an in vitro pull-down assay using recombinant LRB1 and LRB2 proteins, tagged with an N-terminal 6xHis tag as bait, and glutathione *S*-transferase (GST)–HY5 (with an N-terminal GST tag) served as the prey. The GST alone was included as a negative control. GST-HY5 was incubated with LRB1/2-His in two identical reactions and then pulled down using Ni–nitrilotriacetic acid (NTA) beads. In the pellet, we detected GST-HY5, as revealed by immunoblotting using anti-GST antibodies (Fig. 5, A and B), suggesting that both LRB1 and LRB2 physically interact with HY5 in vitro. These results were validated in planta by a BiFC assay in onion epidermal cells. When nYFP-LRB1/cYFP-HY5 and nYFP-LRB2/cYFP-HY5 were coinfiltrated into onion epidermal cells, we could detect a strong YFP fluorescence in the nucleus (Fig. 5C). However, when nYFP-LRB1 or nYFP-LRB2 was coinfiltrated with a cYFP empty vector and cYFP-LRB2 or cYFP-HY5 was infiltrated with nYFP empty vector, YFP fluorescence was not detected (Fig. 5C). The interaction between LRB1 and LRB2 serves as a positive control (*42*).

To further substantiate the physical interaction results, we performed an in vivo Co-IP assay using LRB1-Myc/LRB2-Myc as bait in the *LRB1-OE* and *LRB2-OE* lines. HY5 was detected in the immunoprecipitated fraction of *LRB1-OE* and *LRB2-OE* lines with native anti-HY5 antibody but not in the WT (Fig. 5D). Together, these findings confirm that LRB1/LRB2 physically interact with HY5. To gain further insight into which domain of HY5 is required for the interaction, we generated truncated versions of HY5, HY5N77, and HY5ΔN77 (HY5–C-terminal) clones (Fig. 5E), as described (*64**,* *65*). The GAL4 DBD was fused with HY5N77 and HY5–C terminus to serve as bait, while full-length *LRB1* and *LRB2* were fused with the GAL4 B as prey. The yeast two-hybrid assay revealed that both LRB1 and LRB2 exhibit positive interactions with the HY5–C-terminal domain as the cotransformed colonies could grow on SD-synthetic dropout medium lacking leucine, tryptophan, histidine, and adenine (–LTHA) in the presence of 3-AT (Fig. 5F). These findings confirm that LRB1/LRB2 physically associate with the C-terminal part of HY5, likely resulting in degradation through the 26*S* proteasomal pathway.

### LRBs ubiquitinate and degrade the HY5 transcription factor

**Fig. 6.**
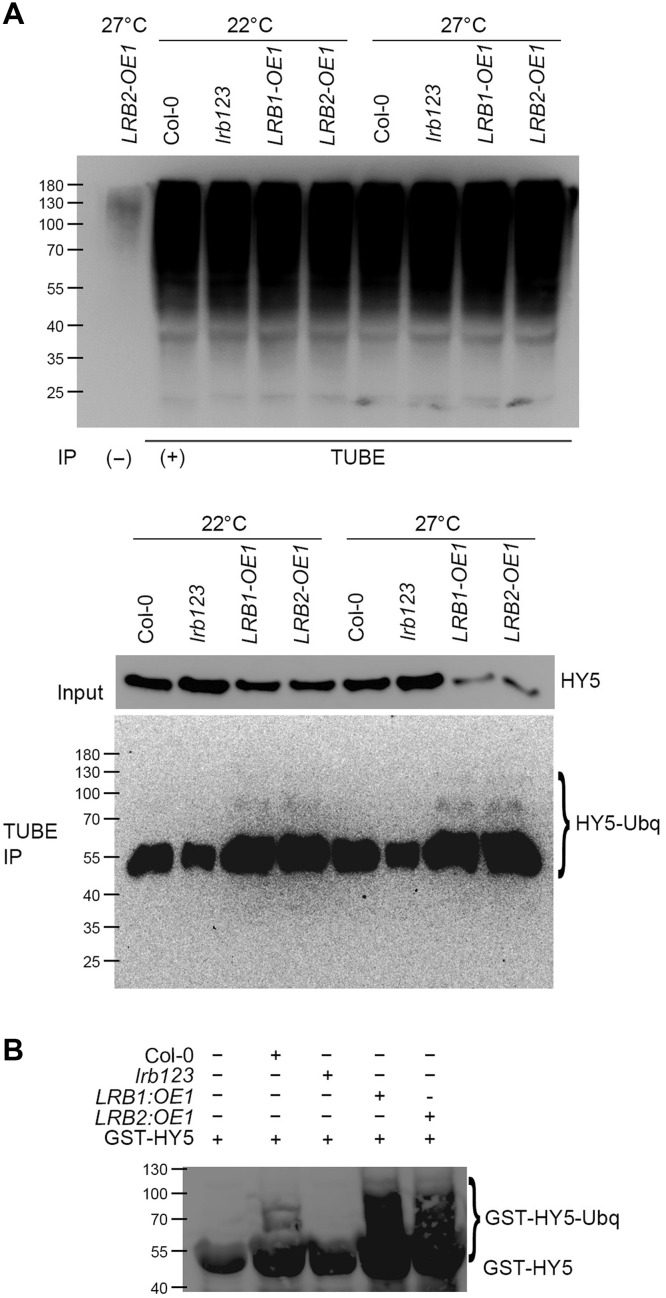
LRBs promote the ubiquitination of HY5 both in vivo and in vitro. (**A**) Immunoblots showing the ubiquitination of HY5 in Col-0, *lrb123*, *LRB1-OE*, and *LRB2-OE* genotypes. Six-day-old seedlings of Col-0, *lrb123*, *LRB1-OE*, and *LRB2-OE*, grown at 22° or 27°C, were incubated in liquid MS supplemented with 80 μM MG132 in the dark at the same temperature for 12 hours before protein extraction. Total ubiquitinated proteins were pooled using TUBE. Immunoprecipitated samples were analyzed by immunoblotting with anti-HY5 or anti-ubiquitin antibodies. (**B**) In vitro ubiquitination assay using seedling extract and recombinant GST-HY5. Purified recombinant GST-HY5 was incubated with seedling protein extracts from Col-0, *lrb123*, *LRB1-OE*, and *LRB2-OE* alongside human ubiquitin-activating enzyme E1 (UBE1), ubiquitin-conjugating enzyme H5c (UbcH5C) (E2), and ubiquitin in ubiquitination buffer. Reactions were carried out at 37°C for 2 hours. Reactions were separated on SDS–polyacrylamide gel electrophoresis (SDS-PAGE) and probed with anti-GST antibody. Increased high–molecular-weight smears represent polyubiquitinated forms of GST-HY5 protein, indicating successful ubiquitination mediated by the E3 ligases in the seedling extract. The first lane without the seedling extract serves as the negative control.

LRBs physically interact and degrade HY5 via the UPS, as seen above. Next, we aimed to investigate whether the LRB-mediated degradation of HY5 is caused by the altered ubiquitination status of HY5. To test this, we used 6-day-old Col-0, *lrb123*, *LRB1-OE*, and *LRB2*-OE seedlings that were grown at 22° and 27°C and treated with MG132 overnight before harvesting the tissue for Co-IP. IP was done for the total ubiquitinated proteins using the tandem ubiquitin binding entities (TUBE) (Fig. 6A, top), and the abundance of ubiquitinated HY5 was detected using anti-HY5 antibodies (Fig. 6A, bottom). The immunoblots suggest that the ubiquitinated form of HY5 (HY5-Ubq) was more accumulated in the LRB overexpression lines compared to Col-0 (Fig. 5A, bottom) at both 22° and 27°C, whereas it is highly reduced in the *lrb123* mutant background.

**Fig. 7.**
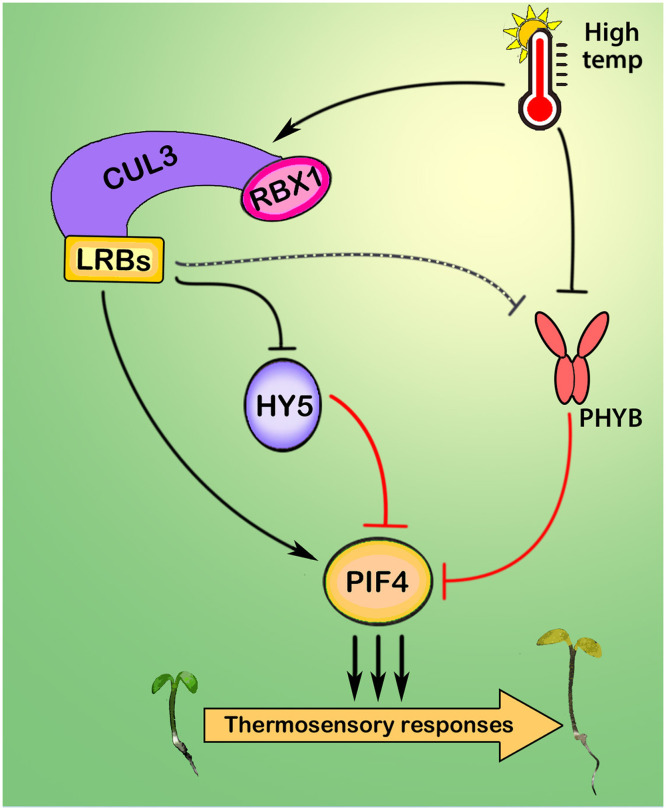
Mechanism of LRB-mediated activation of thermosensory growth. Model illustrating LRB-mediated regulation of thermosensory hypocotyl growth. Elevated temperatures enhance the abundance of LRB1 and LRB2 proteins, thereby increasing PIF4 activity by dampening HY5 activity through the facilitation of its ubiquitination, leading to degradation via the 26*S* proteasomal pathway. Reduced HY5 abundance removes its inhibitory effect on PIF4 by reducing competition at the promoters of PIF4 target genes, thereby enhancing transcription. Concurrently, LRBs also physically associate with PIF4 and prevent its degradation, likely by inhibiting the action of phyB and potential E3 ubiquitin ligases, thereby enhancing PIF4 activity. This dual mode of regulation, in which LRBs promote PIF4 function while inhibiting HY5 activity, ensures optimal thermosensory growth in response to elevated temperatures. RBX1, RING box protein 1.

To further support that LRBs can recognize and promote HY5 ubiquitination, we performed an in vitro ubiquitination assay using a recombinant GST-HY5 fusion protein. Six-day-old seedlings of Col-0, *lrb123*, *LRB1-OE*, and *LRB2*-OE, pretreated overnight with MG132, were harvested and crushed in ubiquitination buffer. Purified GST-HY5 was incubated with E1, E2, ubiquitin, and adenosine 5′-triphosphate (ATP) in lysates prepared from Col-0, *lrb123*, *LRB1-OE*, and *LRB2*-OE seedlings in separate reactions at 37°C for 2 hours. Reactions were stopped and run onto the SDS–polyacrylamide gel electrophoresis (SDS-PAGE). Immunodetection, using the anti-GST antibody, revealed extensively ubiquitinated protein in LRB1-OE and LRB2-OE lysate reactions compared to the WT (Fig. 6B). However, ubiquitination was reduced in the *lrb123* mutant background. These results collectively support the conclusion that LRBs promote the ubiquitination of HY5 protein to facilitate its degradation through the UPS pathway (Fig. 7).

## DISCUSSION

This study reveals LRBs as essential components of the thermosensory pathway required for the temperature-mediated promotion of hypocotyl growth, achieved through the differential regulation of PIF4 and HY5 transcription factor activity. LRB E3 ubiquitin ligases have been shown to negatively regulate photomorphogenic growth in a red- and blue-light–dependent manner (*42*, *46*–*48*). Moreover, LRBs simultaneously target PHYB and PIF3 for degradation (*44*). Our findings unravel LRBs as critical activators of thermomorphogenesis (Fig. 1). Warm ambient temperature–mediated hypocotyl growth is linked to accelerated cell elongation due to increased accumulation of growth hormones such as auxin and BR (*51**,* *57**,* *58*). Genome-wide transcriptome analysis revealed that LRBs are not only required for the expression of genes involved in auxin/BR biosynthesis but also essential for promoting the expression of downstream signaling genes (Figs. 1 and 2) related to growth, cell-wall organization, secondary metabolism, and response to warm temperatures (fig. S6). Moreover, analysis of cellular details, together with pharmacological studies, quantification of endogenous IAA levels, and the *pDR5:GUS* promoter-reporter activity, collectively strongly supports that LRB-mediated thermomorphogenic growth is primarily linked to the enhanced accumulation of plant growth hormones, auxin and BR (Fig. 2 and fig. S3, C and D). Notably enough, a set of light-inducible genes that are repressed by warm temperature treatment is strongly derepressed in the *lrb123* mutant (fig. S9), suggesting that, in addition to promoting the expression of various genes involved in promoting growth, LRBs also repress light-signaling genes involved in photomorphogenesis and pigment accumulation in response to warm temperature. This suggests a dual mode of regulation, whereby LRBs, on the one hand, activate the thermosensory pathway. On the other hand, they suppress the light signaling pathway, leading to robust growth in response to warm temperatures.

PIF4 is a central signaling hub of warm ambient temperature–induced architectural adaptations (*13**,* *18**,* *59*). Genetic interaction studies with *PIF4-OE* and *lrb123* suggest that LRBs are required for PIF4 function (Fig. 3). Genome-wide transcriptome data strongly reveal that many genes differentially regulated in *lrb123* are also differentially expressed in the *pif4* mutant background in the same fashion (fig. S5, C and D). Specifically, genes up-regulated in both *lrb123* and *pif4* showed a strong correlation (*r* = 0.8) (Fig. 3C), suggesting that LRBs overlap with PIF4 and likely function in the same linear pathway. Supporting this, immunoblot analyses showed that PIF4 protein accumulation is markedly reduced in the *lrb123* mutant at both 22° and 27°C (fig. S7) and in response to short-term warmth (Fig. 3E), indicating that LRBs promote thermosensory growth by enhancing PIF4 protein accumulation. The reduced *PIF4* transcript levels in *lrb123* at 27°C but not at 22°C (Fig. 3D) suggest that LRBs regulate *PIF4* expression specifically under warm conditions, likely due to HY5-mediated repression on *PIF4* gene expression as revealed by RT-qPCR results (fig. S13). At 22°C, *PIF4* transcript levels are similar to Col-0, yet protein levels are lower, implying that LRBs also act posttranslationally to maintain PIF4 stability. This stabilization is likely mediated by direct physical interactions between PIF4 and LRB1/2, as shown by multiple protein-protein interaction assays (fig. S10). Consistent with the reduced PIF4 protein levels in *lrb123* and *lrb123 PIF4-OE* plants (Fig. 3 and figs. S7 and S10A), we propose that LRBs protect PIF4 from degradation, plausibly by limiting phyB-mediated turnover and potentially other E3 ubiquitin ligases. Given that phyB promotes PIF4 degradation (*50*) and LRBs target phyB for degradation (*44*), LRBs likely promote PIF4 protein accumulation by reducing phyB-dependent proteolysis. E3 ubiquitin ligases are also known to stabilize transcription factors through noncanonical roles. For example, E3 ligase COP1 not only regulates PIF4 transcription but also physically interacts with and stabilizes PIF4 protein at the posttranslational level (*27*). The COP1/SPA1 complex similarly interacts with and stabilizes PIF3 and PIF5 to promote seedling hypocotyl elongation (*61**,* *66*). COP1 also stabilizes ETHYLENE-INSENSITIVE3 (EIN3) and ETHYLENE-INSENSITIVE3-LIKE1 (EIL1), facilitating seedling emergence from soil (*67**,* *68*). Likewise, the E3 ligases RING DOMAIN OF UNKNOWN FUNCTION 1117 1 (RDUF1) and RDUF2 physically interact with and stabilize HY5 to promote seedling photomorphogenesis (*63*). Together, these examples support a model in which LRB1/2, as E3 ubiquitin ligases, can stabilize the transcription factor PIF4 while simultaneously targeting HY5 for degradation.

It has been reported that *PIF4* gene expression and protein accumulation are strongly induced in response to warm temperatures (*13**,* *27**,* *69**,* *70*), which promotes auxin/BR biosynthesis and signaling (*17**,* *60*). Specifically, auxin biosynthetic and signaling genes, such as *YUC8*, *CYP79B2*, *SAUR19*, and *XTR7*, as well as BR biosynthetic and signaling genes, including *CPD*, *PRE1*, and *PRE5*, show significantly reduced transcript levels in the *lrb123* mutant compared to the WT (Fig. 1G). In our pharmacological studies with picloram and eBL, *lrb12* and *lrb123* mutants fail to recover completely with auxin/BR application, suggesting their key role in auxin/BR signaling pathways in addition to biosynthesis (Fig. 2). Notably, genes such as *LONG HYPOCOTYL IN FAR-RED LIGHT1* (*HFR1*), *HECATE 1* (*HEC1*), and *HEC2*, which are direct targets of PIF4, are also down-regulated in *lrb123*, such as in *pif4* (Fig. 1F). HFR1 and HEC1/2 are part of the negative feedback regulatory loop of PIF4 for fine-tuning its function and optimizing growth (*71**,* *72*). In addition, *HOOKLESS1* (*HLS1*), a direct target of PIF4, cooperates to promote seedling thermomorphogenic growth (*73**,* *74*). Notably, *HLS1* expression was down-regulated in the *lrb123* mutant (Fig. 1F), suggesting that the reduced *HLS1* expression is likely due to reduced PIF4 activity. Together, we propose that LRBs possibly control PIF4 function by promoting its gene expression and protein accumulation and are previously unknown upstream regulators of PIF4, similar to COP1, DET1, and SPA proteins (*18*, *26*, *28*).

In parallel, taking cues from genome-wide transcriptome analysis, our data revealed that LRBs promote thermomorphogenesis simultaneously by enhancing PIF4 activity while inhibiting photomorphogenic responses. Increased light signaling has been shown to dampen the temperature responses (*18*, *28*). Many light-inducible genes repressed by warm temperatures are derepressed in the *lrb123* triple mutant (Fig. 4C and fig. S9D). Many of the photomorphogenesis-promoting genes derepressed in the *lrb123* mutant are regulated by HY5. HY5 is a potent inhibitor of thermomorphogenesis (*18*, *28*, *75*, *76*). HY5 inhibits *PIF4* gene expression and also competes with PIF4 for binding to the same cis-elements on the promoters of growth-responsive genes, thereby inhibiting their expression (*18*, *28*, *75*, *77*). Consistent with this, our genetic and gene expression data suggested that the *hy5* mutant could strongly suppress the *lrb123* mutant growth and gene expression phenotype (Fig. 4). Genetic interaction studies with *hy5* and *lrb123* mutants indicated that reduced temperature response in *lrb123* is likely due to increased HY5 activity, which results in reduced *PIF4* gene expression and enhanced inhibition of PIF4 transcriptional activity at the promoters of growth-responsive genes (figs. S12 and S13). Consistent with this, our immunoblot data confirmed that HY5 stability is substantially elevated in the *lrb123* mutant, especially in the daytime, compared to Col-0 (Fig. 4E). Moreover, cell-free degradation assays also suggest that LRBs likely destabilize HY5 (Fig. 4F). The cell-free degradation results and the in vivo ubiquitination data indicate that LRBs ubiquitinate and degrade HY5 through the 26*S* proteasomal pathway (Figs. 4F and 6). COP1 physically interacts with HY5 through the N-terminal part (HY5N77), degrading it mainly at night (*37**,* *64*). However, our study demonstrates that LRBs interact with HY5 through the C-terminal part (HY5ΔN77) and degrade it under both light and dark conditions. These observations suggest that LRBs likely predominate at 22°C in degrading HY5 during the day, whereas COP1 has a stronger effect in the dark. However, at 27°C, in addition to inhibiting HY5 accumulation during the day, they also destabilize HY5 at night, indicating that LRB1/2 has overlapping and discrete mechanisms to COP1 in inhibiting HY5 function. Together, our study demonstrates that LRBs promote PIF4 activity through two complementary mechanisms: first, by ubiquitinating and degrading HY5, and second, by directly associating with PIF4 to enhance its protein stability (Fig. 7). Through these mechanisms, LRBs potentiate thermosensory growth and gene expression, highlighting their emerging role in integrating light and temperature cues to regulate plant growth and development.

## MATERIALS AND METHODS

### Plant materials and growth conditions

All the mutants and the transgenic lines used in this study are in the background of the Col-0 ecotype of *Arabidopsis thaliana* (*N70000*)*.* The mutant lines *lrb1* (SALK_145146), *lrb2-1* (SALK_001013), *lrb2-4* (N2103780), and *lrb3-1* (SALK_082868) were acquired from the Eurasian Arabidopsis Stock Centre. and *pif4-101* and *hy5-215* were available in laboratory stock. Unless otherwise indicated, *lrb1* refers to the *lrb1-1* allele, *lrb2* refers to the *lrb2-4* allele, and *lrb3* refers to the *lrb3-1* allele. The *pDR5:GUS* (*70*) and the *PIF4-OE* (*pPIF4:PIF4:FLAG*) transgenic line were reported elsewhere (*78*).

For regular maintenance, *A. thaliana* was grown under long-day (LD) conditions [16-hour light (~100 photosynthetic photon flux density, PPFD)/8-hour dark] at 22°C and relative humidity (RH) ~ 70% in 7 × 6 cm pots with a mixture of cocopeat, vermiculite, and perlite (4:2:1) supplemented with Hoagland’s solution once a week. For hypocotyl growth experiments and antibiotic selection, seeds were surface sterilized with 70% (v/v) Merck EMSURE ethanol and 0.5% (v/v) Triton X-100 for 10 min, washed twice with 100% ethanol, dried in a laminar hood, and plated on MS supplemented with 1% sucrose, 0.05% MES, and 0.8 agar as a solidifying agent. Seeds were stratified in the dark for 4 days at 4°C and then transferred to Percival growth cabinets at 22°C under LD conditions for 2 days for germination. Plates were then transferred to the indicated experimental conditions.

### Generation of double and higher-order mutants

*lrb1lrb2* was generated by crossing *lrb1* and *lrb2*, and *lrb1lrb2lrb3* was generated by crossing *lrb1lrb2* with *lrb3*. *lrb123 hy5* was generated by crossing *lrb1lrb2lrb3* with *hy5*. Homozygous lines were confirmed by genotyping via PCR using primers listed in table S1.

### Generation of *lrb123 PIF4-OE*, *LRB1-OE PIF4-OE*, and *LRB2-OE PIF4-OE* lines

*lrb123 PIF4-OE* was generate by crossing PIF4-OE with *lrb1lrb2lrb3*. Double transgenic lines of *LRB1-OE1 PIF4-OE*, *LRB1-OE2 PIF4-OE*, *LRB2-OE1 PIF4-OE*, and *LRB2-OE2 PIF4-OE* were generated by crossing *PIF4-OE* with *LRB1-OE1*, *LRB1-OE2*, *LRB2-OE1*, and *LRB2-OE2*, respectively, followed by selection on phosphinothricin and PCR confirmation using 35*S* primer for *LRB-OEs*.

### Generation of *DR5:GUS lrb123* promoter*-*reporter line

*lrb123 DR5:GUS* was generated by crossing *lrb123* with *DR5:GUS* (Col-0), followed by subsequent phenotypic screening for *lrb123* (short hypocotyl) and GUS staining till a stable population is obtained (F_4_). The line homozygous for transgene and *lrb123* mutations was used for further analysis.

### Generation of *LRB1* and *LRB2* overexpression lines

The *LRB1-OE* and *LRB2-OE* lines were generated as follows. RNA was isolated from Col-0 seedlings, and cDNA was prepared by RT-PCR. Full-length coding sequences of *LRB1* and *LRB2* were amplified by the PCR method using specific primers in table S1. PCR products were cloned into the pENTR Directional TOPO vector as per the manufacturer’s protocol (Invitrogen, #K2400) using One Shot TOP10 *E. coli* cells (Invitrogen, #C404010). Subsequently, both sequences were subcloned into the pGWB-617 *(att*R1-Cm^r^-*ccd*B-*att*R2-4xMyc-T_NOS_) gateway binary vector (*79*) using the LR Clonase II enzyme (Invitrogen). Resultant binary constructs were introduced into *Agrobacterium tumefaciens* (GV3101) and transformed into Col-0 plants via the floral-dip method. Surviving transgenic-T_1_ plants were selected on MS plates containing phosphinothricin (10 μg/ml). Approximately 25 independent lines from the T2 generation were screened for single locus transferred DNA insertion, and two independent homozygous lines were obtained in the T_3_ generation for each transgenic (*LRB1-OE1*, *LRB1-OE2*, *LRB2-OE1*, and *LRB2-OE2*). The higher-order mutants were generated by crossing these lines, followed by confirmation via genotyping using primers listed in table S1.

### Pharmacological assays

For pharmacological assays, seeds were surface sterilized with 70% (v/v) Merck EMSURE ethanol and 0.5% (v/v) Triton X-100 for 10 min, washed twice with 100% ethanol, dried in a laminar hood, and plated on full-strength MS (1% sucrose, 0.05% MES, and 0.8 agar) supplemented with indicated hormones. Picloram [5 μM in dimethyl sulfoxide (DMSO)] was used for auxin response. Epibrassinolide (100 nM in EtOH) was used for BR response, and gibberellic acid (GA_3_; 5 μM in EtOH) was used as gibberellin with respective mocks. Seeds were stratified in the dark at 4°C for 4 days. Plates were then transferred to LD light conditions at 22°C for 1 day to promote germination and subsequently transferred to either 22° or 27°C for an additional 5 days before alignment.

### Histomorphometry studies for cell length measurement

Six-day-old seedlings, grown under SD conditions at either 22° or 27°C, were collected and cleared by incubating in serial dilutions of ethanol (15, 50, 70, 90, and 100%) for 15 min each, progressively. The submerged seedlings, in 100% alcohol, were kept at 4°C overnight. The cleared seedlings were then rehydrated in the reverse order of dilution (100, 90, 70, 50, and 15%) in a similar manner (15 min each) and washed with 0.2 M sodium phosphate buffer (pH 7). For staining, seedlings were dipped in a 1:20 (v/v) dilution of aniline blue stock solution [stock: 0.1% (w/v) of aniline blue (Sigma-Aldrich, Germany) in 0.2 M sodium phosphate buffer] in 0.2 M sodium phosphate buffer and incubated overnight at room temperature. For microscopy, seedlings were mounted onto chloral hydrate solution (chloral hydrate:glycerol:Milli-Q water; 8:1:2) and sealed with a coverslip on top. Samples were examined under an Olympus IX81 inverted microscope equipped with a CoolSNAP MYO CCD Camera and a 40× objective lens. Images were acquired with cellSens Standard Software (Olympus). Hypocotyl cell lengths were defined as the average of cortical cells crossing the middle portion of hypocotyls—the number of cells varies with the genotype and the growth condition. Only the cells extending in the nondividing files were counted. The measurements were taken using ImageJ, and statistical significance was evaluated using the Student’s *t* test with GraphPad.

### RNA extraction and RT-qPCR analysis

For gene expression analysis, seedlings were grown as indicated. Approximately 100 mg of seedlings was crushed in liquid nitrogen using micropestles, and total RNA was extracted using the RNeasy Plant Mini Kit (QIAGEN). A total of 1 μg of RNA was converted to first-strand cDNA using a High-Capacity Reverse Transcription Kit (Applied Biosystems, Thermo Fisher Scientific). Primers used for qPCR have been listed in table S1.

### RNA-seq and differential gene expression analysis

The quality of total RNA was assessed using an Agilent RNA 6000 Nano chip in a 2100 Bioanalyzer (Agilent), and quantitation was performed using a NanoDrop spectrophotometer (Thermo Fisher Scientific), followed by the PicoGreen method in a Qubit fluorometer (Invitrogen). Total RNA samples with high RNA integrity number were selected for sequencing library preparation using KAPA mRNA HyperPrep Kit (KR1352; v5.17) according to the manufacturer’s instructions. The quality of RNA-Seq libraries was checked using high-sensitivity D1000 ScreenTape in an Agilent 2200 TapeStation system, and final library quantification was done using Real-Time PCR (QuantStudio 7 Flex). Paired-end (100 × 2 base pairs) sequencing of these libraries was performed in NovaSeq 6000 (Illumina). Bulk RNA sequence data were then analyzed using standard practices. Briefly, quality control was performed using FastQC, and the reads were mapped to the *A. thaliana* (TAIR10) reference genome (https://plants.ensembl.org/Arabidopsis_thaliana/Info/Index) using HISAT2 to analyze the dataset*.* Subsequently, the mapped sequences were converted from SAM to BAM file format and sorted using the SAMtools package (http://www.htslib.org). Thereafter, the fragments per kilobase of transcript per million mapped reads (FPKM) value was calculated using the StringTie package (https://ccb.jhu.edu/software/stringtie). Following that, the DESeq2 package (https://www.bioconductor.org) was used to perform differential expression analysis between the groups. A *P*_adj_ value cutoff of ≤0.05 and log_2_ fold change cutoff (≤ −1 or ≥ +1) were applied to identify significant DEGs (list of DEGs given in table S1). The heatmaps representing differential gene expression were generated using the R program. The GO enrichment analysis to identify significantly enriched biological process terms in different sets of DEGs was performed using the BiNGO plugin of Cytoscape (v3.9.1) with a cutoff *P* value of <0.05 (*80*). The significantly enriched GO terms were represented via a bubble plot. The comparative GO enrichment plots were generated using the EnrichmentMap tool, which is implemented in Cytoscape.

### Phytohormone quantification

Growth phytohormones were extracted as described in (*81*) with modifications. A total of 25 mg of each lyophilized plant sample was extracted with 1 ml of cold extraction buffer (MeOH:H_2_O:HCOOH; 15:4:0.1) consisting of D2-IAA as internal standard. The homogenized sample was centrifuged at 10,000*g* for 10 min at 4°C. The supernatant was removed and loaded onto a Strata-X (Phenomenex) C18 solid-phase extraction (SPE) column, which had been preconditioned with 1 ml of methanol and 1 ml of 0.1% formic acid in water. After loading, the SPE column was washed twice with a 0.1% formic acid and 5% methanol solution. The sample was eluted with 1 ml of 0.1% formic acid in acetonitrile and dried in a speed-vac. The dried sample was resuspended in 100 μl of 5% methanol and analyzed by liquid chromatography with a SCIEX 6500+ triple-quadruple trap tandem mass spectrometry (MS/MS). Liquid chromatography (LC)-MS/MS was performed according to a published protocol (*82*) with slight modifications. In brief, phytohormone was separated with a ZORBAX Eclipse XDB-C18 column (50 × 4.6 mm, 1.8 μm; Agilent Technologies). The mobile phase comprised solvent A (water and 0.05% formic acid) and solvent B (acetonitrile) with the following elution profile: 0 to 0.5 min, 95% A; 0.5 to 5 min, 5 to 31.5% B in A; 5.01 to 6.5 min, 100% B; and 6.51 to 9 min, 95% A, with a flow rate of 1.1 ml/min. The column temperature was maintained at 25°C. For detection, the mass spectrometer was operated in positive ionization mode [multiple reaction monitoring (MRM) modus] to monitor analyte parent ion → product ion transition as described (*83*). Settings were as follows: ion spray voltage: 5500 eV; turbo gas temperature: 650°C; nebulizing gas: 70 psi; curtain gas: 45 psi; heating gas, 60 psi. The Analyst 1.5 software (Applied Biosystems) was used for data acquisition and processing.

### GUS histochemical assay

The histochemical assay was performed as described in (*84*) with modifications. Six-day-old seedlings were fixed in 0.3% formaldehyde, 10 mM MES (pH 5.6), and 0.3 M mannitol for 1 hour at room temperature after a brief (1 min) vacuum. Seedlings were washed five times in 50 mM NaH_2_PO_4_ (pH 7.0) and incubated in assay buffer (1 mM X-Gluc in 50 mM NaH_2_PO_4_, pH 7.0) at 37°C overnight after a brief vacuum. After staining, seedlings were rinsed in 70% ethanol for 20 min and then mounted for microscopy.

### GUS fluorometry assay

Six-day-old WT and *lrb123* mutant seedlings expressing β-glucuronidase reporter enzyme under *DR5* gene promoter (*pDR5:GUS*) were harvested. A total of 100 mg of the tissue was crushed in liquid N_2_ and homogenized in extraction buffer [50 mM phosphate buffer (pH 7.0), 1 mM Na_2_EDTA, 10 mM dithiothreitol (DTT), 0.1% sodium lauryl sarcosine, and 0.1% Triton X-100], centrifuged at 15000 rpm for 15 min at 4°C, and the debris were separated. A total of 50 μl of these extracts was then incubated in 950 μl of assay buffer [1 mM methylumbelliferyl-\(\beta \)-D-glucuronide (MUG) in extraction buffer], mixed thoroughly, and incubated overnight. The reaction was stopped with a stop buffer (0.2 M Na_2_CO_3_ in H_2_O). The fluorimeter was calibrated with freshly prepared MU standards, and fluorescence was measured with excitation at 365 nm and emission at 455 nm.

### Protein extraction and immunoblotting

Six-day-old seedlings (100 mg) were harvested in 2 ml of microcentrifuge tubes, frozen, and crushed in liquid nitrogen and ground using micro pestles in 300 μl of extraction buffer [00 mM tris-Cl (pH 7.5), 100 mM NaCl, 20% (v/v) glycerol, 5 μM phenylmethylsulfonyl fluoride (PMSF), 20 μM β-mercaptoethanol, 20 μM DTT, and 1× protease inhibitor cocktail (Roche)]. The tubes were centrifuged at 15,000 rpm for 15 min to pellet the debris, and the supernatant was collected in fresh tubes. A total of 5 μl of the extract was used for protein estimation by Bradford’s assay. For the rest of the protein extract, an appropriate volume of 6× sample buffer [350 mM tris-HCl (pH 6.8), 8% SDS (m/v), 50% glycerol (v/v), 20% (v/v) β-mercaptoethanol, and 0.05% bromophenol blue] was added and boiled for 10 min at 90°C. The protein was separated on 10% SDS-PAGE and transferred to a polyvinylidene difluoride membrane. Primary antibodies—Rabbit polyclonal antibody against PHYB (Agrisera, AS214566), Rabbit polyclonal antibody against HY5 (Agrisera, AS121867), Goat polyclonal antibody against PIF4 (Agrisera, AS163955), and Rabbit polyclonal antibody against ACTIN (Sigma-Aldrich, SAB4301137)—and secondary antibodies (immunoglobulin G–horseradish peroxidase)—Goat anti-rabbit antibody (Abcam, ab205718) and Rabbit anti-goat antibody (Invitrogen, 81-1620)—were used. Immunoblots were developed using the SuperSignal West Femto Substrate (Thermo Fisher Scientific, 34096).

### Cell-free degradation assay

For the purification of recombinant HY5-His, the full-length coding sequence of the HY5 gene was amplified and cloned into pET-20b(+) to create a translational fusion construct with a His tag. The tagged proteins were expressed in *E. coli* strain BL21 (DE3) and purified with Ni-NTA agarose beads (QIAGEN, 30210). For the cell-free degradation assay, seedlings were harvested and ground into fine powder in liquid nitrogen. The proteins were extracted in degradation buffer [25 mM tris-HCl (pH 7.5), 10 mM NaCl, 10 mM MgCl2, and 10 mM ATP] and centrifuged at 15000 rpm for 15 min to remove the debris. Protein concentration was estimated using Bradford’s assay and adjusted to equal concentration for both the *lrb123* mutant and the WT (Col-0) with degradation buffer. For each reaction, 40 ng of recombinant HY5-HIS protein was incubated with 160 ng of extract. A mouse monoclonal antibody against the His tag (Invitrogen, HIS.H8) was used for immunodetection.

### In vitro pull-down assay

A DNA fragment encoding full-length HY5 was cloned into the pGEX-4 T2 vector to generate translational fusion constructs with an N-terminal GST tag. DNA fragments encoding full-length LRB1 and LRB2 were cloned into the pET28a(+) vector to get the translational fusion constructs with the N-terminal His tag using primers listed in table S1. Recombinant GST-HY5, His-LRB1, and His-LRB2 proteins were expressed and purified from *E. coli* strain BL21 (DE3). GST-HY5 was purified by glutathione agarose beads (GoldBio, G-250). His-LRB1 and His-LRB2 were purified by Ni-NTA agarose beads (QIAGEN, 30210).

For in vitro binding experiments, His-LRB1 or His-LRB2 (1.5 μg each) proteins were incubated with 50 μl of Ni-NTA agarose beads in the pull-down buffer [50 mM tris-Cl (pH 8), 150 mM NaCl, 0.2% (v/v) glycerol, 1 mM EDTA, 1 mM PMSF, 0.1% (v/v) Nonidet P-40, and 2× protease inhibitors cocktail (Roche)] for 2 hours at 4°C. The beads were centrifuged at 2000 rpm, and the supernatant was removed. Excess unbound protein was washed off by repeating the step five times with 1 ml of pull-down buffer. The beads were incubated with 2% skimmed milk for 1 hour at 4°C and washed again. The equimolar ratio of GST-HY5 or GST protein was added in 500 μl of pull-down buffer and incubated for 2 hours at 4°C. Beads were collected by centrifugation and washed three times with the pull-down buffer. The pellet was resuspended in 25 μl of 6× SDS loading buffer, boiled for 5 min, and analyzed on SDS-PAGE. Rabbit polyclonal antibodies against the GST tag (Bio Bharati Life Sciences, BB-AB0020) were used for immunodetection.

### Yeast two-hybrid assay

For the yeast two-hybrid protein-protein interaction assay, full-length LRB1 and LRB2 were cloned into the pGADT7 vector using the respective primers (table S1) to obtain translational fusion proteins with the AD. Full-length HY5, partial HY5-N77, and HY5ΔN77 were cloned into the pGBKT7 vector to obtain a translational fusion protein with the binding domain. Similarly, full-length LRB2 was also cloned into the pGBKT7 vector using the respective primers listed in table S1. For investigating protein-protein interactions, corresponding plasmids were cotransformed into the yeast strain Y2HGold using Matchmaker Gold Yeast Two-Hybrid System as per the manufacturer’s protocol (Takara, 630489) and plated on double-dropout medium lacking Trp and Leu amino acids. Positive interacting combinations of proteins were identified on quadruple-dropout medium lacking Trp, Leu, His, and Ade, supplemented with 5 mM 3-AT.

### BiFC assay

Full-length coding sequences of HY5, LRB1, and LRB2 were cloned into the pENTR Directional TOPO vector as per the manufacturer’s protocol (Invitrogen, #K2400). Subsequently, LRB1 and LRB2 were subcloned into the pE-SPYNE-778 containing the N-terminal part of YFP, whereas HY5 and LRB2 were subcloned into pE-SPYCE-777 containing the C-terminal part of YFP, both under the control of the 35*S* promoter. These plasmids were then individually transformed into the *A. tumefaciens* (GV3101) using freeze-thaw transformation. The plasmid harboring the *P19* gene of tomato bushy stunt virus, under the control of the 35*S* promoter, was also transformed into *Agrobacterium*.

*Agrobacterium* infiltration in onion scale leaves was carried out as described in (*85*) with modifications*. Agrobacterium* harboring *LRB1* and *LRB2* in *pE-SPYNE-778* and *pE-SPYCE-777* and *HY5* in *pE-SPYCE-777* constructs were cultivated overnight in YEM (0.002% MgSO_4,_ 0.02% KH_2_PO_4_, 0.004% NaCl, 0.1% yeast extract, and 0.25% mannitol) medium supplemented with rifampicin (30 mg/liter) and ampicillin (100 mg/liter), whereas the one harboring the *p19* construct was supplemented with 50 mg/liter of kanamycin and rifampicin. The culture was incubated at 28°C and harvested at optical density at 600 nm (OD_600_) of 0.9 to 1.2 by centrifugation at 5000 rpm for 15 min and resuspended in 10 ml of infiltration medium [41.65 mM d-glucose, 100 mM CaCl2, 100 mM MES-KOH (pH 5.6) stock solution, 0.011 mM 6-benzylaminopurine (BAP), 0.01% Silwet L-77, 0.05 mM MgCl2, and 400 μM acetosyringone]. Centrifugation and resuspension were repeated five times. Last, the cells were diluted to an optical density (OD_600_) of 0.1. Different combinations of cells were prepared by mixing in a 2:2:1 ratio (protein A:protein construct B:*p19*) and injected into the interface between the adaxial epidermis and mesophyll of onion scales, until a bubble for agroinfiltration was observed on the lower side of the onion peel. The injected onion bulbs were incubated in the dark for 72 hours at 24°C. Peel mounts were prepared from the injected part and observed under the epifluorescence microscope (Olympus IX81).

### In vivo ubiquitination assay

Six-day-old seedlings, grown under SD conditions at 22°C, were treated in liquid MS supplemented with 80 μM MG132 overnight (in dark) and then transferred to light for 4 hours at 27°C. Seedlings were soaked on tissue paper and immediately frozen in liquid nitrogen. A total of 200 mg of tissue was ground in 300 μl of Co-IP buffer [100 mM tris-Cl (pH 7.5), 100 mM NaCl, 0.5% (v/v) Nonidet P-40, 5% (v/v) glycerol, 1× protease inhibitor cocktail (Roche), 80 μM MG132, 2 mM PMSF, 20 mM iodoacetamide, pepstatin (1 μg/ml), aprotinin (2 μg/ml), and leupeptin (5 μg/ml)]. IP was done using TUBE 2 (FLAG) (LifeSensors, #UM602) and FLAG affinity beads (Bio Bharati Life Sciences, #BB-FL001B). In a 1.5-ml microcentrifuge tube, 15 μg of TUBE 2 (FLAG) (LifeSensors, #UM602) was incubated overnight with 120 μl of DYKDDDDK affinity beads in 200 μl of Co-IP buffer at 4°C on a rocker. Beads were washed twice with wash buffer [100 mM tris-Cl (pH 7.5), 100 mM NaCl, and 0.5% (v/v) Nonidet P-40]. The extracted protein was then incubated overnight with the beads at 4°C. The supernatant was removed, and the beads were washed thrice with the wash buffer. The beads were resuspended in 25 μl of elution buffer [100 mM tris-Cl (pH 7.5), 200 mM NaCl, and 0.5% (v/v) Nonidet P-40). A total of 5 μl of 6× Laemmli buffer was added and incubated at 70°C for 10 min before loading onto SDS-PAGE. Anti-HY5 (Agrisera) and anti-ubiquitin (Invitrogen) were used for immunoblotting.

### In vitro ubiquitination assay

Human ubiquitin-activating enzyme E1 (UBE1), ubiquitin-conjugating enzyme H5c (UbcH5C)-His, and biotinylated ubiquitin were obtained from Abcam. For the HY5 ubiquitination assay, affinity-purified GST-HY5 fusion protein was subjected to further purification by size-exclusion chromatography. Gel filtration was performed using a Superdex 200 pg preparative SEC column (Cytiva) installed on an ÄKTA start FPLC system (Cytiva). The gel filtration buffer consisted of 50 mM tris-HCl (pH 8.0), 150 mM NaCl, 5% (v/v) glycerol, and 2 mM dithiothreitol (DTT). All purification steps were carried out at 4°C. For plant extracts, 250 mg of 6-day-old seedlings was harvested in liquid nitrogen and crushed in 100 μl of ubiquitination buffer (Abcam) with the addition of inorganic pyrophosphatase (IPP) (100 μl/ml), 50 mM EDTA, 50 mM DTT, and 80 mM MG132. Ubiquitination reactions were set up by incubating 250 nM E1, 5 μM E2, 500 nM HY5, 50 μM biotinylated ubiquitin, and 5 mM ATP into the plant extracts prepared from the indicated genotypes for 2 hours at 37°C. Reactions were stopped with 6× loading buffer. HY5 was detected by Western blot analysis using an anti-GST antibody.

### Phenotypic measurements and statistical analysis

For phenotypic measurements, images were taken using a Canon EOS M50. Seedlings were aligned on charcoal plates (0.8% agar and 2.5% charcoal powder) and imaged from the top for hypocotyl measurements. For statistical analysis, one-way analysis of variance (ANOVA) was followed by Tukey’s post hoc test as described. Measurements were done using ImageJ (https://imagej.net/ij/), and graphs were plotted using GraphPad Prism.

## Supplementary Materials

### The PDF file includes:

Figs. S1 to S13 Legends for datasets S1 and S2 Legend for table S1

### Other Supplementary Material for this manuscript includes the following:

Datasets S1 and S2
