# Supplementary material for: CUL3LRB E3 ubiquitin ligases control thermosensory growth in Arabidopsis by differentially regulating HY5 and PIF4 protein stability
Source: Sci Adv. 2026 Mar 6;12(10):eaec7817. doi: 10.1126/sciadv.aec7817 (PMC12965291; doi:10.1126/sciadv.aec7817)
Supplement: Supplementary file 1 — Figs. S1 to S13 Legends for datasets S1 and S2 Legend for table S1 [file sciadv.aec7817_sm.pdf]

Science Advances
AAAS

# Supplementary Materials for

## CUL3$^{LRB}$ E3 ubiquitin ligases control thermosensory growth in *Arabidopsis* by differentially regulating HY5 and PIF4 protein stability

Chirag Singhal *et al.*

Corresponding author: Sreeramaiah N. Gangappa, ngsreeram@iiserkol.ac.in

*Sci. Adv.* **12**, eaec7817 (2026)
DOI: 10.1126/sciadv.aec7817

**The PDF file includes:**

Figs. S1 to S13
Legends for datasets S1 and S2
Legend for table S1

**Other Supplementary Material for this manuscript includes the following:**

Datasets S1 and S2
Table S1

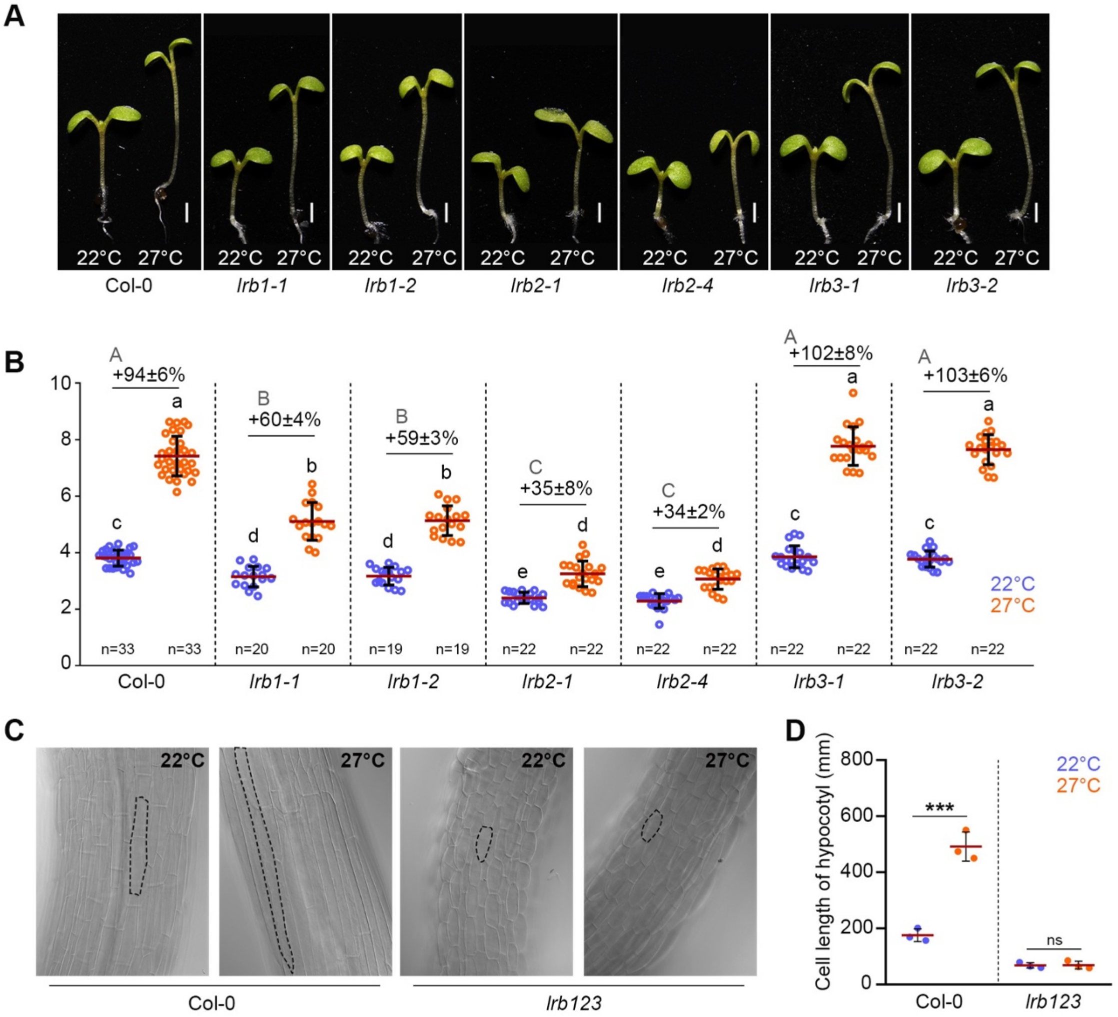


**Fig. S1. LRBs exhibit a non-redundant but additive phenotype in response to temperature.**

(**A** and **B**) Representative seedling images (A) and the hypocotyl length measurement data (B) of various mutants of *LRB1, LRB2 and LRB3. The lrb1* and *lrb2* have short hypocotyls, while *lrb3* does not display much difference in the hypocotyl lengths compared to Col-0. Six-day-old seedlings of the WT (Col-0), *lrb1-1*, *lrb1-2*, *lrb2-1, lrb2-4, lrb3-1, lrb3-2* and Col-0 are shown in (A) (scale bar: 2 mm). (B) Normalized hypocotyl length (27°C/22°C) in Col-0, *lrb1*, *lrb2* and *lrb3* mutants. Different small letters denote that the groups are significantly different from one another (One-way ANOVA followed by Tukey's HSD post-hoc test, $p<0.05$). "*n*" indicates the number of seedlings measured. Seeds were stratified for four days and then allowed to germinate for a day at 22°C before being transferred to 22°C or 27°C under SD photoperiod.

(**C**) Differential interference contrast images of hypocotyls through the middle portion of Col-0 and the *lrb123* mutant.

(**D**) Cell length measurements from the middle portion of the hypocotyl. The entire hypocotyl length of the seedlings was developed by end-to-end stitching of the individual frames. 70 per cent of the hypocotyl length from the middle was considered for cellular measurements. Selectively, non-dividing, elongated cells in ($n>3$) seedlings were measured for statistical analysis. $n \geq 45$ cells were measured in Col-0_22ºC, $n \geq 30$ in *lrb123*_22ºC, $n \geq 30$ in Col-0_27ºC, $n \geq 45$ in *lrb123*_27ºC in each respective genotype. Asterisks indicate significance after Student's t-test (***$P < 0.001$).

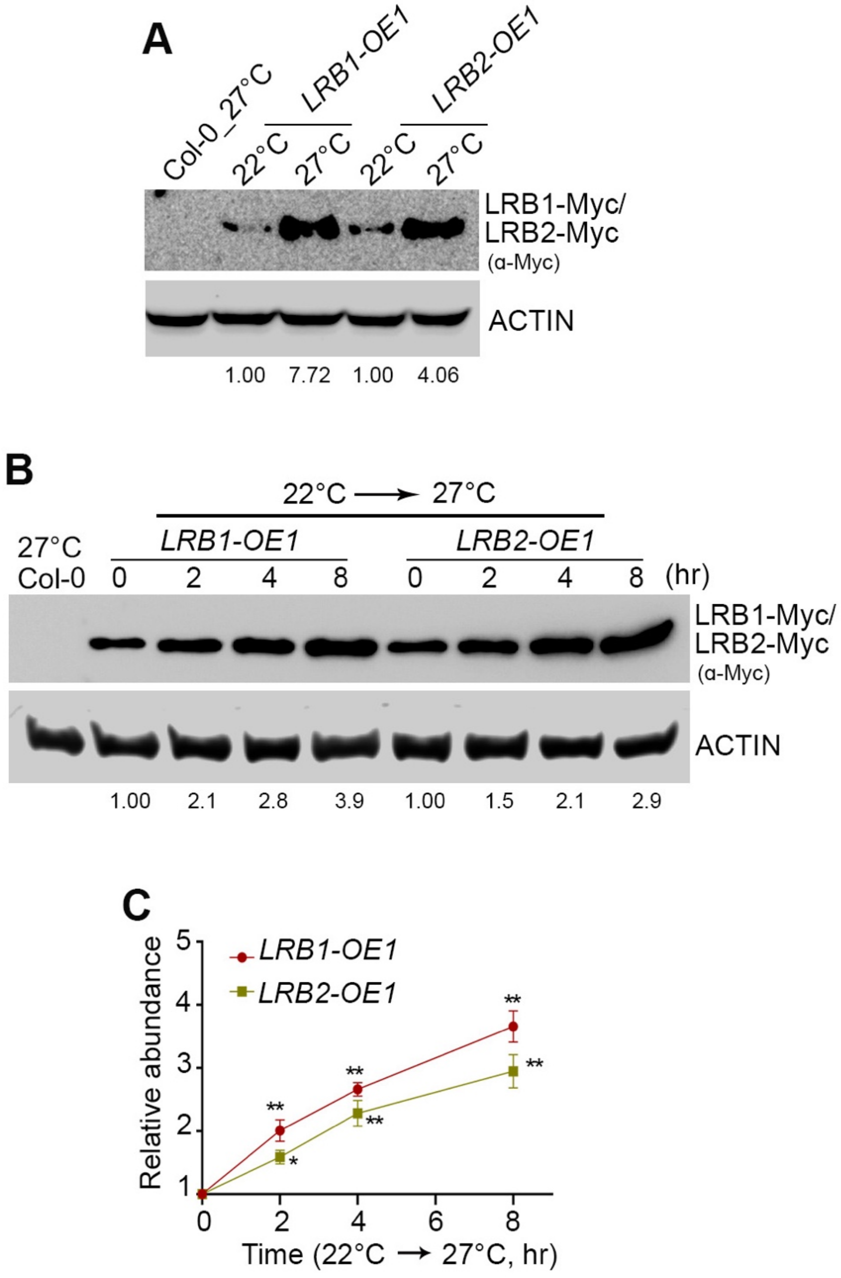


**Fig. S2. LRB1/LRB2 protein abundance is enhanced at warm temperatures.** (**A**) LRB1-Myc and LRB2-Myc protein abundance in the six-day-old seedlings. Immunoblots showing LRB1-Myc or LRB2-Myc protein levels in the wild type (WT) and *lrb123* mutant grown at 22ºC or 27ºC for six days. Numbers below the

immunoblots indicate the relative intensities of the bands. Actin was used as the loading control.

(**B** and **C**) Relative protein abundance of LRB1-Myc and LRB2-Myc proteins in six-day-old seedlings. (**B**) Immunoblots showing LRB1-Myc and LRB2-Myc protein levels in the *pLRB1:LRB1:Myc* and *pLRB2:LRB2:Myc* grown at 22ºC and then shifted to 27ºC. ACTIN is used as a loading control. Numbers below the immunoblots indicate the relative intensities of the bands. (**C**) Relative PIF4 protein abundance in Col-0 and *lrb123* mutant. Points on the line graph show the mean, and the error bar shows the standard deviation of three replicates. For the immunoblot experiment in B, seedlings were grown and harvested at the indicated time points after being transferred to 27°C on the sixth day. Asterisks denote statistical significance at the protein level at the 0 time point in Col-0. The relative intensity of LRB1-Myc/ACTIN or LRB2-Myc/ACTIN is calculated and normalized by the same at the 0h time point in the respective lines after transferring to 27ºC, as mentioned below the immunoblots. Asterisks in the graph indicate the significance level as revealed by the Student's *t*-test: *$P < 0.05$; **$P < 0.01$.

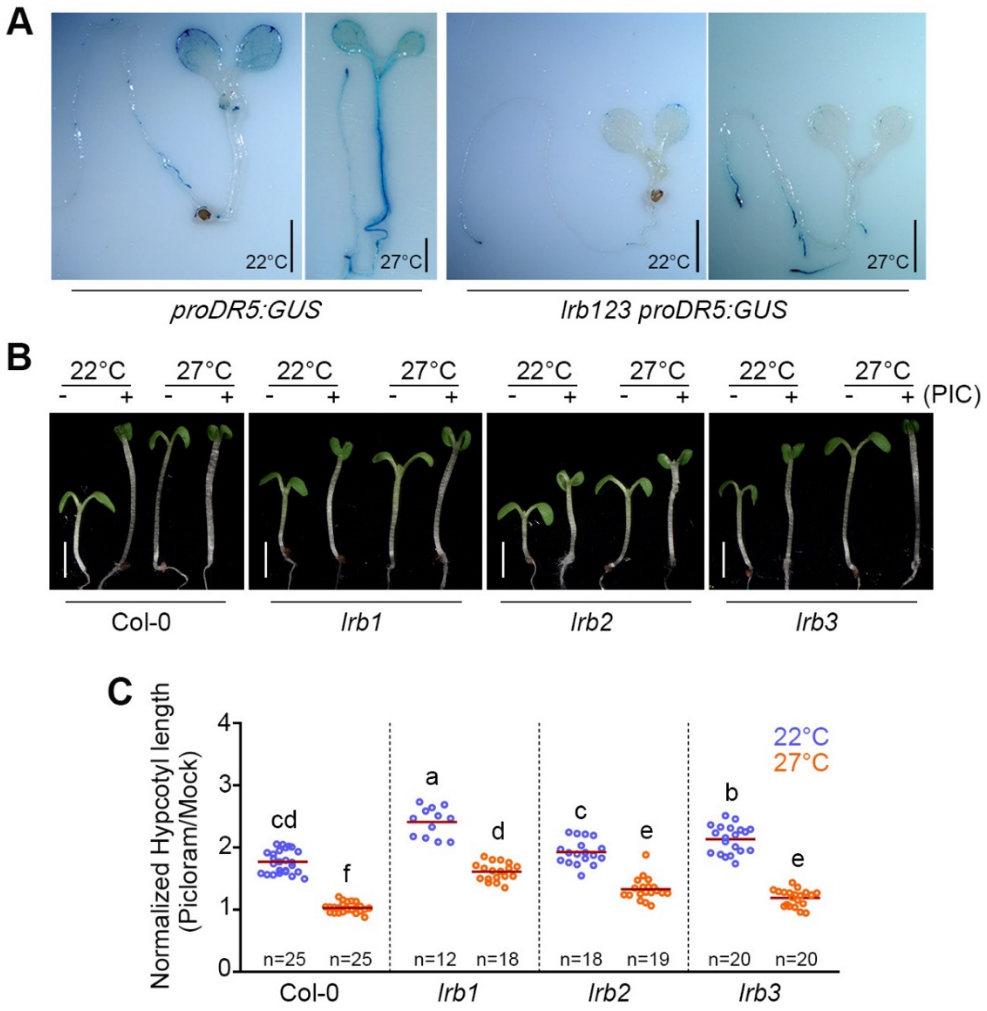


**Fig. S3. Exogenous application of auxins rescues the short hypocotyl phenotype of *lrb1* and *lrb2* mutants.**

(**A**) Auxin reporter *proDR5:GUS* expression in Col-0 and *lrb123* mutant background. The *proDR5:GUS* activity in six-day-old seedlings of Col-0 and *lrb123* at optimum (22°C) and warm temperature treatment (27°C) (scale bar: 2 mm). Approximately 20 seedlings for each genotype were used for histochemical staining.

(**B**) Six-day-old seedlings of Col-0, *lrb1*, *lrb2* and *lrb3* grown in the presence or absence (DMSO as mock) of 5 µM of picloram at either 22°C or 27°C (scale bar: 2 mm). (**C**) Normalized hypocotyl length (picloram/mock) in Col-0, *lrb12,* and *lrb123* mutants. Seedlings from two independent experiments were measured. Different small letters denote that the normalized hypocotyl lengths are significantly

different ($p<0.05$, after one-way ANOVA followed by Tukey’s HSD post-hoc test). “*n”* indicates the number of seedlings measured.

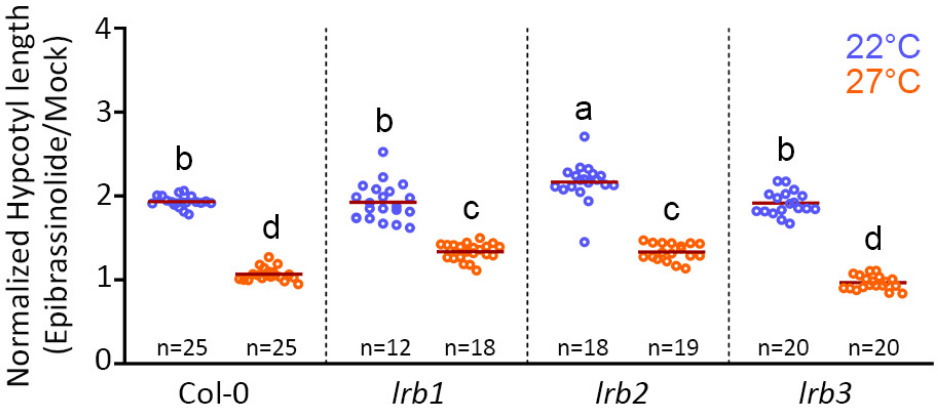


**Fig. S4. Exogenous application of brassinosteroids rescues the short hypocotyl phenotype of *lrb1* and *lrb2* mutants.**

Normalized hypocotyl length (epibrassinolide/mock) in Col-0, *lrb1, lrb2* and *lrb3* mutants. Seedlings from two independent experiments were measured. Different small letters denote that the normalized hypocotyl lengths are significantly different ($p<0.05$, after one-way ANOVA followed by Tukey's HSD post-hoc test). "*n*" indicates the number of seedlings measured.

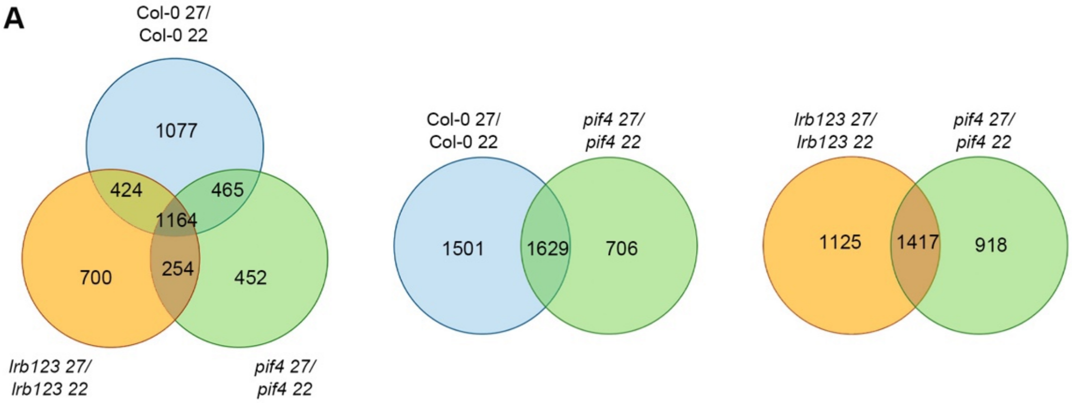


**B**

| Comparisons / Differential Expression | Col-0 vs. *lrb123* vs. *pif4* | Col-0 vs. *lrb123* | Col-0 vs. *pif4* | *lrb123* vs. *pif4* | Col-0 | *lrb123* | *pif4* |
|---|---|---|---|---|---|---|---|
| upregulated | 688 | 183 | 244 | 198 | 534 | 380 | 336 |
| downregulated | 476 | 241 | 221 | 56 | 543 | 320 | 116 |
| total | 1164 | 424 | 465 | 254 | 1077 | 700 | 452 |

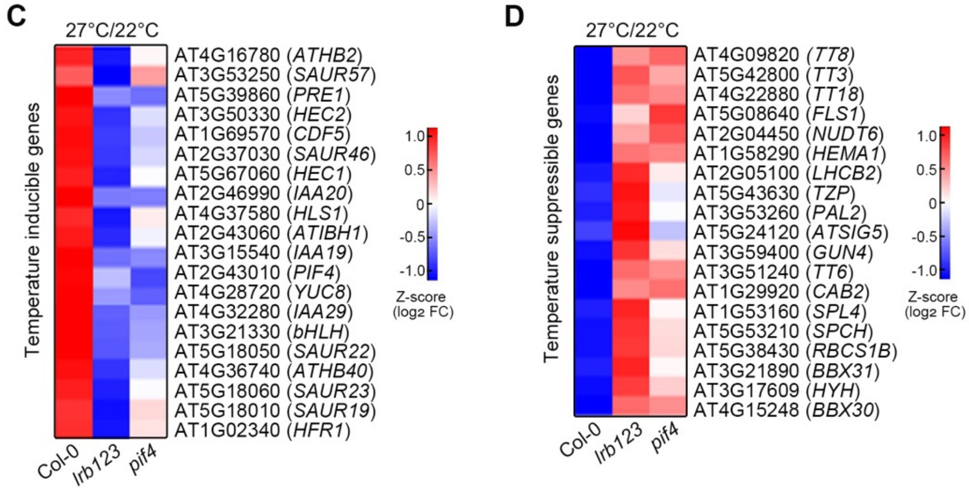


**Fig. S5. Transcriptional profiling of Col-0, *lrb123* and *pif4* mutants.**

(**A**) Differentially regulated genes shared among Col-0, *lrb123* and *pif4* mutant seedlings in response to warm temperature (27°C/22°C).

(**B**) A comparative analysis table shows the differentially expressed genes in different groups.

(**C** and **D**) Temperature-responsive genes in *lrb123* and *pif4* mutants exhibit a similar pattern of misregulation compared to those in Col-0 in response to warm temperatures. Selected temperature-induced (**C**) and temperature-suppressed (**D**)

genes are shown via heat maps. A cut-off of $\log_2$ fold-change $\geq 1$ or $<-1$ with a $P<0.05$ was used to identify differentially expressed genes (DEGs). Three biological replicates were analyzed for each genotype.

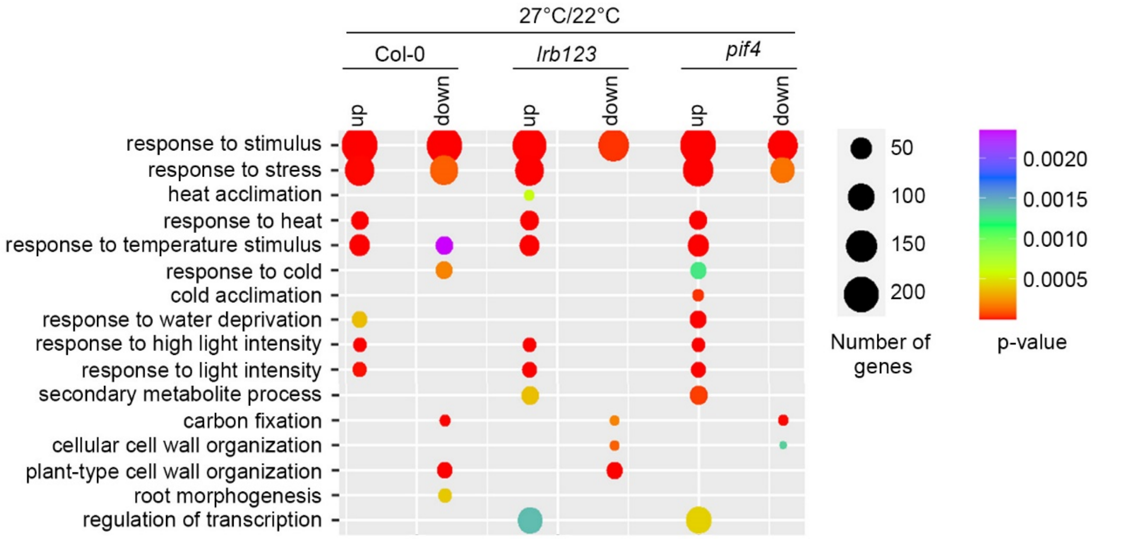


**Fig. S6. GO term enrichment analysis of DEGs in response to 27°C compared to 22°C (27°C/22°C) in Col-0, *lrb123* and *pif4* mutants.**

Comparative GO term analysis in the sets of up- and down-regulated genes identified in the different comparisons via the EnrichmentMap plug-in implemented in Cytoscape (v3.7.1). The significance level and the number of genes for each GO term were plotted via a bubble plot. The bubble size denotes the number of genes misregulated in an enriched term, and the colour of the bubble marks the significance in terms of *p*-value. GO terms with *P*-value ≤0.05 were considered to be significantly enriched. The GO enrichment analysis to identify significantly enriched biological process terms in different sets of DEGs was performed using the Bingo plugin of Cytoscape (v3.9.1) with a cut-off *P*-value of <0.05 (*85*)

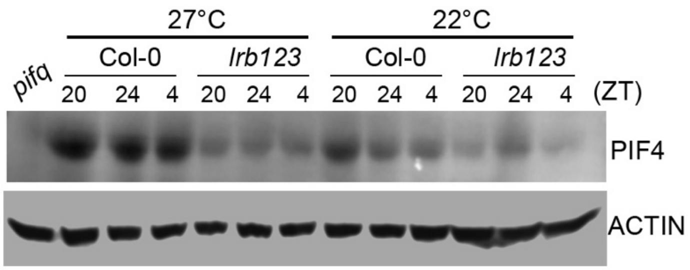


**Fig. S7. LRBs promote PIF4 protein accumulation.**
(Immunoblots showing endogenous PIF4 protein levels in the wild type (WT) and *lrb123* mutant grown at 22ºC and 27ºC. Tissues were harvested for protein extraction at the mentioned time points. Anti-PIF4 antibody was used to detect PIF4. ACTIN levels serve as the loading control.

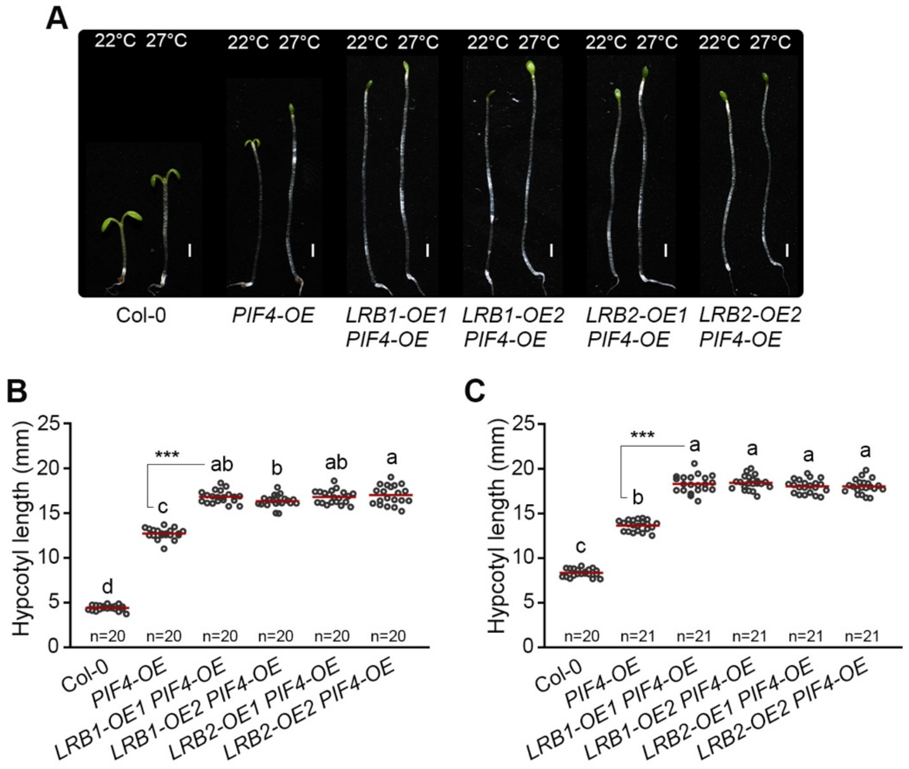


**Fig. S8. Overexpression of *LRB1* and *LRB2* further enhances the *PIF4-OE* line hypocotyl phenotype.**

(**A**) Representative images of six-day-old seedlings of Col-0 and *LRB1-OE1 PIF4-OE*, *LRB1-OE2 PIF-OE*, *LRB2-OE1 PIF4-OE* and *LRB2-OE2 PIF4-OE* double transgenic lines. Scale bar: 1 mm.

(**B** and **C**) Hypocotyl length of Col-0, *LRB1-OE1 PIF4-OE*, *LRB1-OE2 PIF-OE*, *LRB2-OE1 PIF4-OE* and *LRB2-OE2 PIF4-OE* seedlings grown at 22°C (B) and 27°C (C) under short-day photoperiod. Seedlings from two independent experiments were measured. In B and C, different small letters denote that the groups are significantly different from one another (One-way ANOVA followed by Tukey's HSD post-hoc test, $p<0.05$). "*n*" indicates the number of individual seedlings measured.

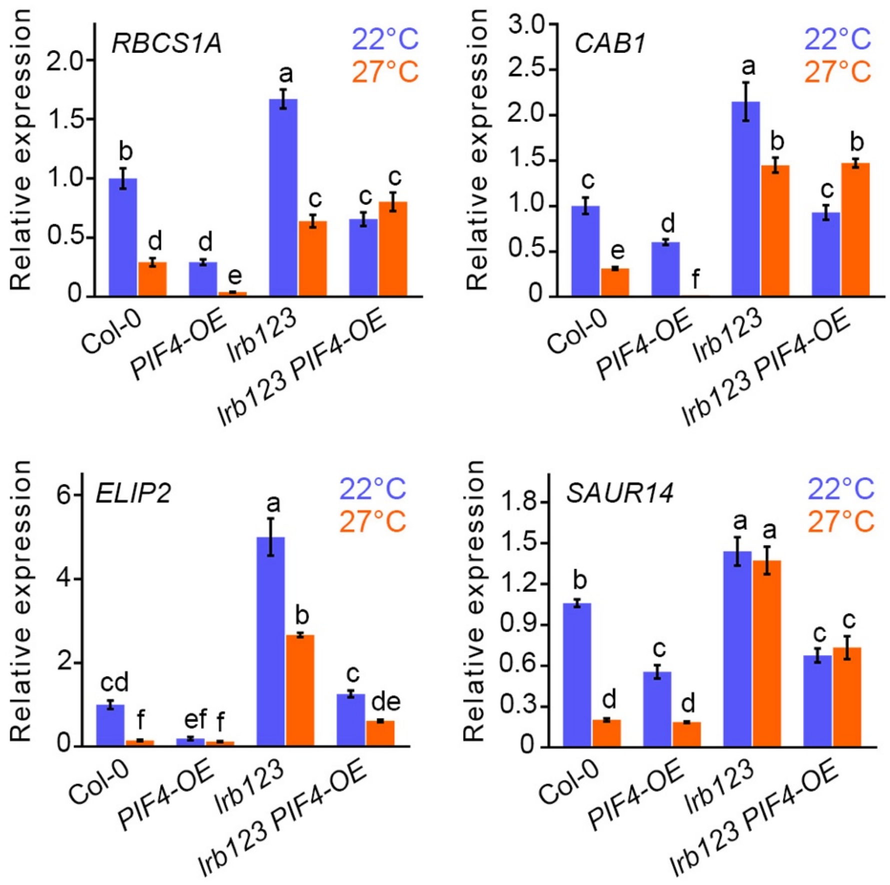


**Fig. S9 Reduced expression of light-responsive genes in *PIF4-OE* are derepressed under the *lrb123* mutant background.**

Relative expression of temperature repressible genes measured through RT-qPCR in six-day-old seedlings of Col-0, *lrb123*, *PIF4-OE* and *lrb123 PIF4-OE* genotypes. *EF1α* was used as an endogenous control. Expression of genes in Col-0 at 22°C is used for normalization. The data represent the mean with error bars indicating ±SD ($n = 3$ biological replicates). Small letters above the bars denote that the groups are significantly different from one another, as revealed by one-way ANOVA followed by Tukey's HSD post-hoc test ($p<0.05$).

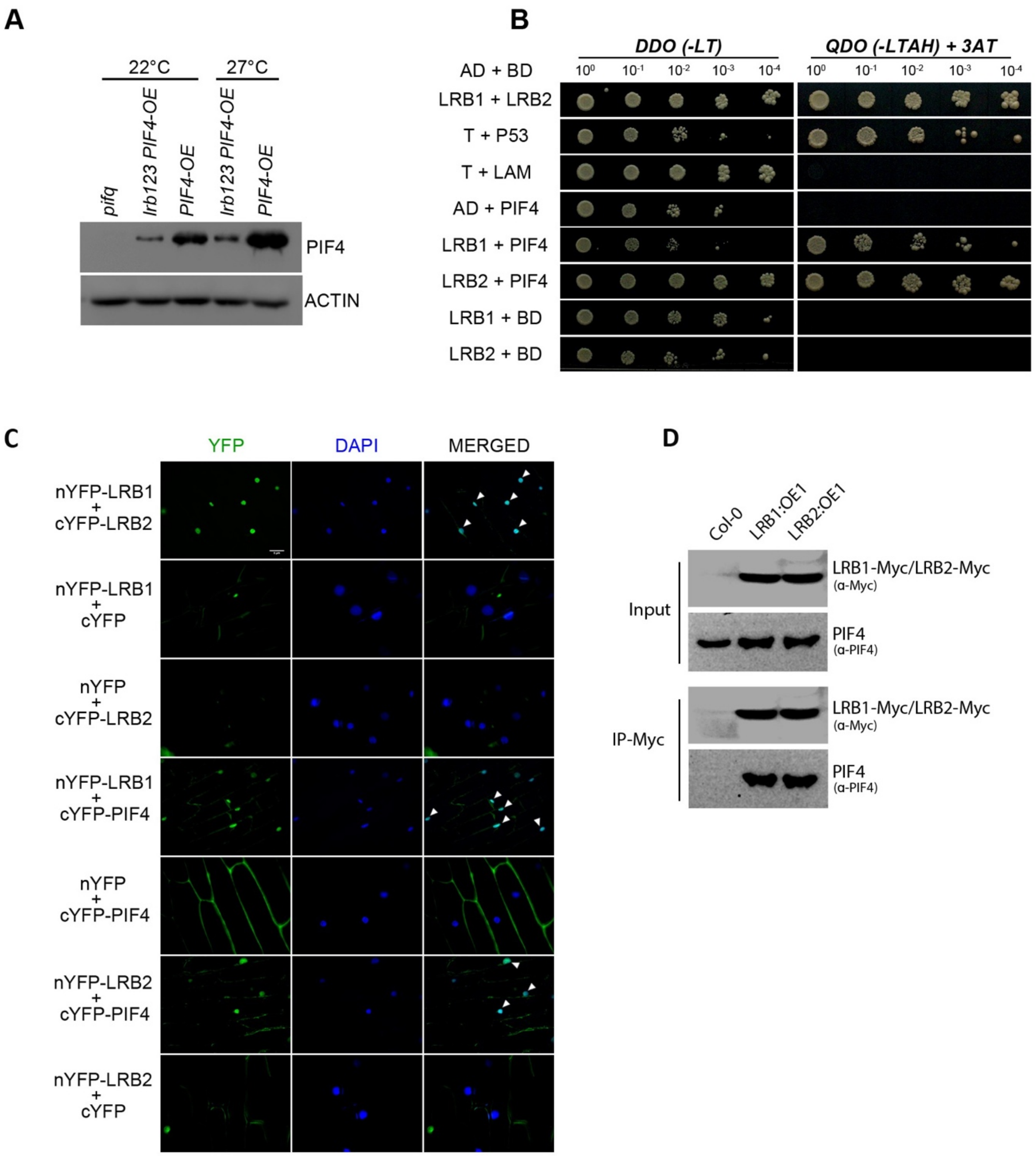


**Fig. S10. LRBs promote PIF4 protein accumulation likely through direct physical association.**

(**A**) Immunoblots showing endogenous PIF4 protein levels in the *PIF4-OE* and *lrb123 PIF4-OE* lines grown at 22ºC and 27ºC. Tissues were harvested for protein extraction at ZT8. Anti-PIF4 antibody was used to detect PIF4. ACTIN levels serve as the loading control.

(**B**) Yeast two-hybrid assay showing interactions of LRB1 and LRB2 with PIF4. The left panel shows the growth of the co-transformed yeast Y2H-Gold strain on double-dropout media, and the right panel shows the growth on quadruple-dropout media supplemented with 5 mM 3-AT.
(**C**) Epi-fluorescence microscopy images showing BiFC signals of the indicated protein pairs transiently expressed in *Allium cepa* epidermal cells. Hoechst 33342 was used to stain the nuclei (green), and GFP (red) signals are shown. Scale bar: 5 μm. The weak cytoplasmic YFP signal observed in some controls is background fluorescence.
(**D**) Immunoblots showing co-immunoprecipitation assay of PIF4 protein with LRB1-Myc and LRB2-Myc. LRB1-Myc/LRB2-Myc were immunoprecipitated using anti-Myc antibody. An anti-PIF4 antibody was used to detect co-immunoprecipitated PIF4. Col-0 is used as a negative control.

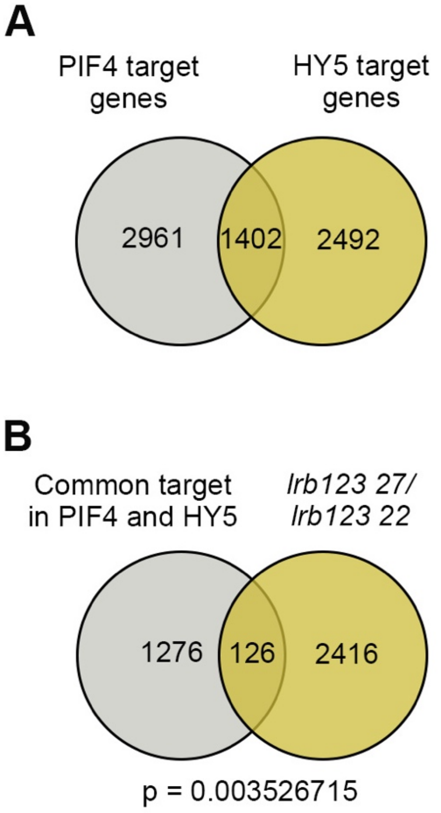


**Fig. S11. A set of genes targeted by both HY5 and PIF4 also shows misregulation in the *lrb123* mutant in response to warm temperatures.**
(**A**) Overlapping genes among the sets of target genes of HY5 (*36*) and PIF4 (*62*).
(**B**) Overlapping genes among target genes of HY5 (*1*) and misregulated genes in the *lrb123* mutant in response to temperature (22°C/27°C).

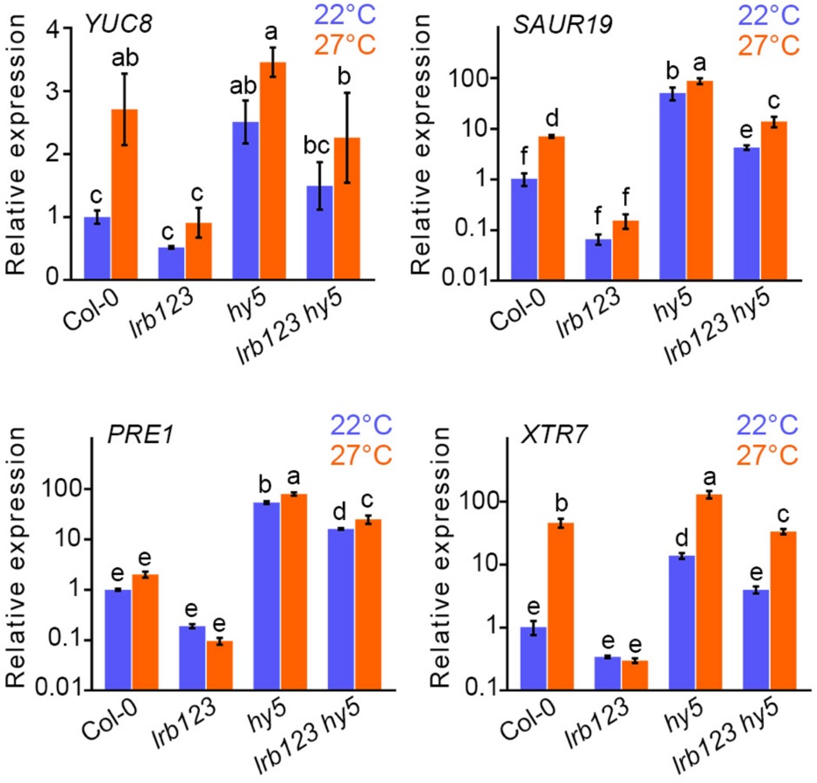


**Fig. S12. The *hy5* mutation derepresses the growth-related genes under the *lrb123* mutant background.**

Relative expression of temperature-inducible genes measured through RT-qPCR in six-day-old seedlings of Col-0, *hy5*, *lrb123* and *lrb123 hy5*. *EF1α* was used as an endogenous control. Expression of genes in Col-0 at 22°C is used for normalization.

The data are presented as the mean with error bars indicating ±SD ($n = 3$ biological replicates). Small letters above the bars denote that the groups are significantly different from one another, as revealed by one-way ANOVA followed by Tukey's HSD post-hoc test ($p<0.05$).

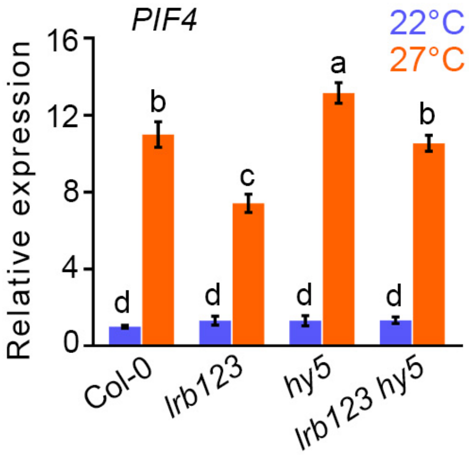


**Fig. S13. Reduced *PIF4* gene expression in the *lrb123 hy5* quadruple mutant.** Examining the *PIF4* gene expression in six-day-old seedlings of Col-0, *lrb123*, *hy5* and *lrb123 hy5* genotypes in 22°C and 27°C under short-day photoperiod. *EF1α* was used as an endogenous control. The expression of *PIF4* in Col-0 at 22°C is used for normalization. The data are presented as the mean±SD (*n=3* biological replicates). Small letters above the bars denote that the groups are significantly different from one another, as revealed by one-way ANOVA followed by Tukey's HSD post-hoc test ($p<0.05$).

**Other Supplementary Materials include the following (uploaded as separate files):**

**Dataset S1.** List of differentially expressed genes in Col-0, *lrb123* and *pif4* genotypes from six-day-old seedlings grown at 22°C and 27°C.

**Dataset S2.** Gene ontology analysis of differentially expressed genes in Col-0, *lrb123* and *pif4* genotypes in response to warm temperature (27°C).

**Tables S1.** List of Oligonucleotides used in this study.
